# Microscale Electrochemical Corrosion of Uranium Oxide Particles

**DOI:** 10.3390/mi14091727

**Published:** 2023-09-01

**Authors:** Jiyoung Son, Shawn L. Riechers, Xiao-Ying Yu

**Affiliations:** 1 Energy and Environment Directorate, Pacific Northwest National Laboratory, Richland, WA 99354, USA; 2 Oak Ridge National Laboratory, Materials Science and Technology Division, Oak Ridge, TN 37830, USA

**Keywords:** uranium oxide (UO_2_), particle-attached electrode, microscale electrochemical cell, multimodal characterization, Nafion membrane, system for analysis at the liquid–vacuum interface (SALVI)

## Abstract

Understanding the corrosion of spent nuclear fuel is important for the development of long-term storage solutions. However, the risk of radiation contamination presents challenges for experimental analysis. Adapted from the system for analysis at the liquid–vacuum interface (SALVI), we developed a miniaturized uranium oxide (UO_2_)-attached working electrode (WE) to reduce contamination risk. To protect UO_2_ particles in a miniatured electrochemical cell, a thin layer of Nafion was formed on the surface. Atomic force microscopy (AFM) shows a dense layer of UO_2_ particles and indicates their participation in electrochemical reactions. Particles remain intact on the electrode surface with slight redistribution. X-ray photoelectron spectroscopy (XPS) reveals a difference in the distribution of U(IV), U(V), and U(VI) between pristine and corroded UO_2_ electrodes. The presence of U(V)/U(VI) on the corroded electrode surface demonstrates that electrochemically driven UO_2_ oxidation can be studied using these cells. Our observations of U(V) in the micro-electrode due to the selective semi-permeability of Nafion suggest that interfacial water plays a key role, potentially simulating a water-lean scenario in fuel storage conditions. This novel approach offers analytical reproducibility, design flexibility, a small footprint, and a low irradiation dose, while separating the α-effect. This approach provides a valuable microscale electrochemical platform for spent fuel corrosion studies with minimal radiological materials and the potential for diverse configurations.

## 1. Introduction

The safety and security of spent fuel in long-term storage is a critical issue due to the complexity of the material in terms of its chemical composition and physical properties. The radiological level of spent fuel is often significant and will take several thousands of years to decay to a level close to natural uranium ore [1]. One of the most widely anticipated long-term storage solutions for spent fuel is the deep geological repository (DGR) method because it offers the best passive safety system for permanent disposal. The internationally accepted DGR design stores spent fuel rods in corrosion-resistant used-fuel containers (UFCs) that can withstand hydrostatic, lithostatic, and glaciation loads. The sealed UFCs are placed at a depth of 500 m or more in a suitably dense intact rock with an additional surrounding bentonite clay barrier [1,2,3,4,5,6,7,8,9,10,11].

Since the repositories are planned to last for thousands of years and more, it is necessary to calculate the radionuclide source term in a DGR and compare the predicted results with those from experiments [1,8]. One of the key perspectives of existing modeling efforts is that the dissolution rate of spent fuel can be strongly influenced by hydrogen (H_2_). Hydrogen can be produced from the radiolysis of groundwater (H_2_O). Also, the production of H_2_ from the canister iron material as a corrosion product in the presence of multiple possible oxidants, such as OH, H_2_O_2_, O_2_, HO_2_, and CO_3_^2−^, is considered a main route for the radiation-induced dissolution of spent fuel [7,8,12,13,14]. This scenario can occur when the spent fuel rods are exposed to groundwater via small cracks in the canister. Other scenarios leading to the presence of H_2_O in a DGR are due to the saturation of spent fuel rods orcooling of the local environment [1,2,15].

There are two major groups of spent fuel geological depository models based on experimental data from different projects, namely the European Commission’s Model Uncertainty for the Mechanism of Dissolution of Spent Fuel in a Nuclear Waste Repository (MICADO) and the Canadian repository program. Based on experimental data from the MICADO project, five models have been developed, including the matrix alteration model (MAM), the Kungl Tekniska Högskolan (KTH) model, the subatomic physics and associated technologies (SUBATECH) model, the French Alternative Energies and Atomic Energy Commission (CEA) model, and another CEA model similar to the SUBATECH model [8,16,17]. Based on the Canadian repository program’s experimental data, two models were developed, namely the mixed potential model (MPM) and the fuel matrix dissolution model (FMDM) [8]. Recently, the U.S. Department of Energy (DOE) has adapted FMDM for the dissolution and degradation of spent fuel waste. The MPM and FMDM account for hydrogen reactions by quantifying their effects on the electrochemical corrosion potential of the fuel. This is accomplished by including hydrogen reactions on both UO_2_ fuel grains and noble metal fission products, like alloy particles, which are also referred to as epsilon phase or noble metal particles [8,18].

Many experiments were performed to study UO_2_ spent fuel corrosion [3,19,20,21,22,23,24,25,26,27,28]. For example, Shoesmith’s group presented UO_2_ corrosion mechanisms and reaction constants to study the α-radiolysis effect under conditions relevant to long-term waste storage using an electrochemical testing setup [24]. Additionally, recent radiolytic modeling efforts used available kinetic data relevant to spent fuel storage conditions [19,22,29]. Of particular interest to the quantification of spent fuel corrosion, X-ray photoelectron spectroscopy (XPS) was used to study surface film formation from the anodic oxidation of polycrystalline UO_2_ in a neutral sodium sulfate (Na_2_SO_4_) solution. The exposed surface area of the bulk UO_2_ electrode was approximately 1.6 cm^2^ using potentiostatic and cyclic voltametric (CV) techniques [25]. They also investigated the spent fuel corrosion by comparing the surface layer and polished sub-layer of the electrically driven corrosion mechanism of UO_2_ using XPS. Salt solutions containing sodium perchlorate (NaClO_4_) and sodium bicarbonate (Na_2_CO_3_) were used as the electrolyte [21].

Previous spent fuel corrosion experiments used a bulk UO_2_-attached working electrode (WE) that had a surface area of several centimeters squared and a thickness in the order of millimeters (mm) (e.g., a 1.6 cm^2^ surface area on the tip of a metal shaft) to reduce the radiological contamination risk while using unirradiated UO_2_ [18,25,30]. However, the radiological contamination risk could not be eliminated using macro-sized electrodes when actual nuclear spent fuel was used with this design and volume. Therefore, conducting experiments on spent fuel is extremely logistically challenging due to the requirements and difficulty in handling radioactive materials, as well as facility limitations [31]. Consequently, hot cells and other radiological facilities are required. Thus, making a UO_2_ electrode of micrometer (µm) size that is compatible with microfluidic platforms [32,33] offers an attractive, innovative solution. Microscale electrochemical cells use less UO_2_ mass in miniaturized electrodes, thus significantly reducing the radiological contamination risk. This can provide a higher degree of freedom to perform experiments, potentially out of hot cells. Furthermore, the inherent miniaturized micrometer (μm)-sized electrode helps to separate the α-effect from β/γ radiolysis.

Our group has developed microfluidic cells for the multimodal spectroscopy and microscopy of liquids [34,35,36], called the system for analysis at the liquid–vacuum interface (SALVI). The electrochemical version of the SALVI cell, or the E-cell, contains three electrodes (working, counter-, or reference electrodes, or WE, CE, and RE), and is integrable to a suite of spectroscopy and imaging techniques for in situ and operando analysis [34,35,36]. Recently, we explored several new techniques based on the direct deposition on an electrode (DDE) to enable spent fuel research using the SALVI E-cell as an established and reliable platform for electrically driven corrosion studies. Specifically, two DDE methods were developed, namely the conductive epoxy-stamping and Nafion spin-coating methods, for the fabrication of UO_2_ analog cerium oxide (CeO_2_) material onto a gold (Au) conductive layer as a WE that is suitable for microscale electrochemical cells [32,33].

Nafion is an ideal material for electrochemical study due to its high proton conductivity, selective permeability to water, and outstanding chemical stability [37,38,39,40]. To increase the physical durability, Nafion is an efficient strategy to protect particles in WE [41,42]. Herein, Nafion was used as a protection layer in the E-cell after attaching UO_2_ particles. The thickness of the Nafion film can be precisely controlled by altering the spinning rate [32].

In this work, we demonstrated that µm-sized electrodes containing UO_2_ particles can provide a novel, alternate solution to study spent fuel corrosion using the modified SALVI E-cell. Miniaturized testing cells can significantly reduce radiological exposure. A smaller amount of radiological materials means that researchers can use low-radiological-risk protocols, perform more tests, and use more than one instrument platform to study the reaction pathways. Fabricated UO_2_-containing electrochemical devices were evaluated to determine analytical reproducibility and electrochemical performance using CV. Atomic force microscopy (AFM) and XPS were used to verify the corrosion effects on the electrode surface and compare the as-made (pristine) and corroded UO_2_ electrodes.

Our results show that microscale electrodes significantly reduce the mass loading of UO_2_ particles in a series of experiments. Additionally, they can provide useful information with minimal radiological contamination risk. Considering the challenges of utilizing UO_2_ as an electrode component with regard to the strict safety protocols of radioactive materials [31], this novel microscale approach will increase the degree of freedom to study more diverse spent fuel corrosion conditions, including synfuels and noble metal particles, in the future.

## 2. Materials and Methods

### 2.1. WE Fabrications and E-Cell Device Assembly

**Figure 1:**
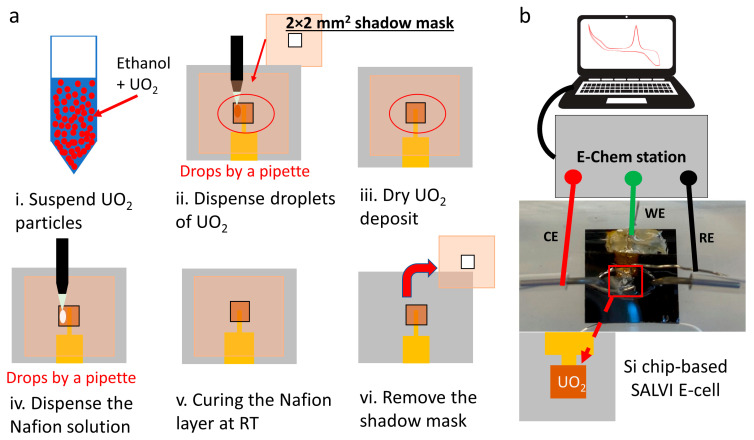
(**a**) The schematic showing the step-by-step fabrication process of particle deposition (i–iii), Nafion film formation (iv–vi); (**b**) the assembled electrochemical device and the setup to perform an electrochemical analysis of UO_2_-containing SALVI E-cells.

Unirradiated single-crystal UO_2_ particles were used to develop methods. A small amount of target UO_2_ particles of 184 mg was suspended in 1 mL of ethanol (Millipore Sigma, 200 proof, ACS reagent, ≥99.5%), as shown in Figure 1a. Ethanol was used for a faster air-drying UO_2_ deposition than DI water. A few droplets of the suspended mixture were pipetted onto the shadow masked Au-WE layer of the Si chip (Figure 1a) [33]. CeO_2_ was used to reduce radiological wastes during method development (Figure S3) [32]. Deposited UO_2_ samples were dried at room temperature (25 °C) for a half day. A drop of Nafion (Sigma Aldrich, St. Louis, MO, USA) was applied onto the UO_2_-deposited WE area. The deposited Nafion was cured at room temperature (25 °C) to form a homogeneously distributed layer [32]. The droplet deposition of Nafion was utilized instead of the spin-coating method, mainly because of the time constraints of setting up a new work procedure in a radiological space [32]. Due to uncured Nafion’s water-like characteristics, it can be uniformly deposited on the wanted surface and uniformly cured (see Figures S1–S3). The thickness of the Nafion layer using the droplet deposition is slightly thicker than that of the spin-coating method. However, this slight difference does not prevent the characterization of the unique UO_2_ WE signal from the slightly higher Nafion background. Figure S2 shows that the overall WE thickness difference is approximately 2 µm between the 500 rpm spin-coated surface and the drop-applied case. This result is consistent with previous profilometer measurements between the spin-coated and the droplet-deposited Nafion WE [32]. Thus, droplet deposition was reasonable for demonstration purposes. The optimal amount of particles was also investigated by testing multiple devices with a duplicate set to reduce the potential contamination risk. The SALVI E-cell uses Pt wires as the CE and the RE. Parts of these devices were fabricated using soft lithography. More details on SALVI fabrication are available in the Supplementary Information section and previous reports [34,36].

### 2.2. Electrochemical Analysis

CV was used to investigate device performance. The electrolyte containing 0.1 M of sodium perchlorate (NaClO_4_) was used following previous works [24]. A series of CV potential scans were performed using two microscale electrochemical cells, namely devices A and B, to verify reproducibility. Figure 1b shows the experimental setup of the SALVI E-cell. The UO_2_ electrochemical cells were evaluated using a CV sweeping start from −1 V to 1 V, then sweeping back from 1 V to −1 V at scan rates of 10, 20, 40, 60, 80, and 100 mV/s, respectively. CV sweeps on the Nafion control SALVI E-cell were also performed to compare with those of UO_2_ particles. The corroded WE chips were retrieved for AFM and XPS analysis. Pristine (as-synthesized) electrode chips with UO_2_ were also characterized to determine the corrosion effects after electrochemical measurements.

### 2.3. AFM Analysis

An MFP-3D Infinity AFM (Asylum, Oxford, Santa Barbara, CA, USA) was used for the topographical analysis of the as-made and corroded WE surfaces. An etched silicon probe (Bruker, RTESPA-300, 8 nm nominal tip radius, 40 N/m spring constant, Billerica, MA, USA) with a set point of 3 V and a scan speed of 0.3 Hz was used for tapping mode measurements.

### 2.4. XPS Analysis

XPS (Kratos, AXIS Ultra DLD, Waltham, MA, USA) analysis of UO_2_ WEs were performed with a monochromatic Al-Ka source (h_n_ = 1486.7 eV) operating at an analysis chamber pressure of <2 × 10^−9^ Torr. Both pristine and corroded WEs were inserted into an anoxic glove box filled with argon that is connected to the fast entry-port of the XPS. Double-sided copper tape (3M^®^, 1182, Taipei, Taiwan) was pressed against the UO_2_ WE surface then slowly peeled off to exfoliate the UO_2_ from the Si chip (see result at Figure S7). This setup enabled the probing of the material that was closest to the Au surface and adjacent to the UO_2_ WE. The Si chip substrate with the UO_2_ WE or with the exfoliated UO_2_ WE substrate was then transferred into the instrument via the load-lock before each XPS analysis.

During the analysis, surface charging was minimized using a low-energy electron flood gun. Survey spectra were acquired at a pass energy (PE) of 160 eV and a step size of 1 eV, while high-resolution data were acquired at a PE of 40 eV with a step size of 0.1 eV. For reference, the Au(4f_7/2_) feature of a sputter-cleaned Au foil yielded a full width at half maximum (FWHM) of 1.9 eV at a PE of 160 eV, while a PE of 40 eV provides a FWHM of 0.8 eV.

The acquired data were processed using the Casa XPS software and were charge-referenced to adventitious C(1s) (C-C/C-H component) at 285.0 eV. Two different methods were used to fit the U(4f) peak to quantify the contribution of each uranium oxide component in the electrodes by considering only the U(4f7/2) line and both the U(4f7/2) and the U(4f5/2), respectively. The first method allows for a simpler peak model with the satellite features observed in the U(4f5/2) region being left out. Since the f-core-level lines in XPS occur at a fixed distance and have fixed area ratios, using both core-level doublets is not always necessary [43]. The second method considers all satellite features along with both the core-level U(4f) peaks. These two methods produced comparable results with a minor difference of 2 At%. The results produced from the second method are presented herein (see Table S3), since all peaks and satellite features are accounted for in the U(4f) narrow-scan spectra.

### 2.5. Surface Tension

A KRUESS K12 Tensiometer (KRUESS, Hamburg, Germany) was used to determine the surface hydrophobicity of the electrodes under different fabrication and corrosion conditions (Table S4a,b). The measurements were used to provide evidence of water incursion into the Nafion membrane. Surface tension was determined using the Wilhelmy plate method. A solution of 0.1 M NaClO_4_ was used to perform triplicate measurements.

### 2.6. Raman

Raman-scattering spectra were recorded with a Horiba LabRAM HR Evolution Raman confocal microscope, using 633 nm laser with no attenuation and a 40× magnification objective. The corroded and pristine electrodes were analyzed in the range from 100 to 4000 cm^−1^.

### 2.7. Beta and Gamma Particle Count from the UO_2_ WE

Radiological survey was conducted following an established protocol. A handheld beta-gamma detector (Model 44-9 Ludlum, Sweetwater, TX, USA) and an alpha-beta-gamma scaler (Ludlum 2929 Ludlum, Sweetwater, TX, USA) were utilized to make measurements. The β- and γ particle counts were measured for each WE to determine their radiation level.

## 3. Results and Discussion

### 3.1. Studying Electrochemically Driven UO_2_ Redox Reactions Using Microscale E-Cells

**Figure 2:**
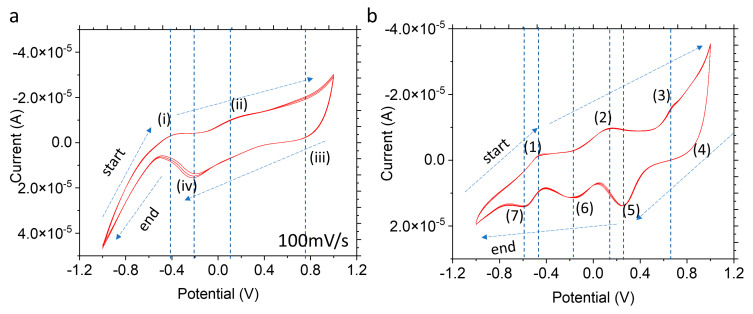
CV results of (**a**) UO_2_ WE (Device A) and (**b**) Nafion control WE with a 100 mV/s scan rate in 0.1 M of NaClO_4_ electrolyte.

The UO_2_ device’s fabrication reliability was demonstrated. Figure 2a depicts CV results of device A, with a scan rate of 100 mV/s. The result shows a unique UO_2_ CV profile compared to the Nafion control in Figure 2b. During the oxidation process of the UO_2_ WE device from −1 V to 1 V, the observed prominent anodic peak potentials were found at −0.4 V (marked as process (i)) and 0.13 V (process (ii)), respectively (Figure 2a). The anodic peak (i) may represent the oxidation process of UO_2_/U(V) to UO_3_, 2UO_2_ + O_2_ → 2UO_3_ or U_3_O_8_ + 6H_2_O_2_ → 3UO_3_ + 6H_2_O [24,44,45]. Peak (ii) with a peak potential of 0.13 V (process (2)) appears to correspond with anodic peaks from the Nafion control WE profile (Figure 2b), which slightly shifted to the right.

During potential sweeps of the reduction process (form 1 V to −1 V) of the UO_2_ WE device, prominent cathodic peaks were observed at −0.24 V (process (iv)) and 0.81 V (process (iii)), respectively. The peaks observed at −0.24 V (iv) vs. platinum (Pt) RE could be due to the reduction of U (V) to UO_2_ by the following: U_3_O_8_ → 3UO_2_ + O_2_ [46,47,48]. Peak (iii) may correspond to the cathodic peak (4) from the reduction in the Nafion control device with a peak potential 0.83 V, which slightly shifted to the right. In comparison, the CV profiles of the Nafion control device shows four peaks at −0.60 V (process (7)), −0.17 V (process (6)), 0.23 V (process (5)), and 0.83 V (process (4)), which likely occur due to surface reductions. These anodic and cathodic peaks require further analysis for more accurate reaction pathway interpretation in future studies.

**Figure 3:**
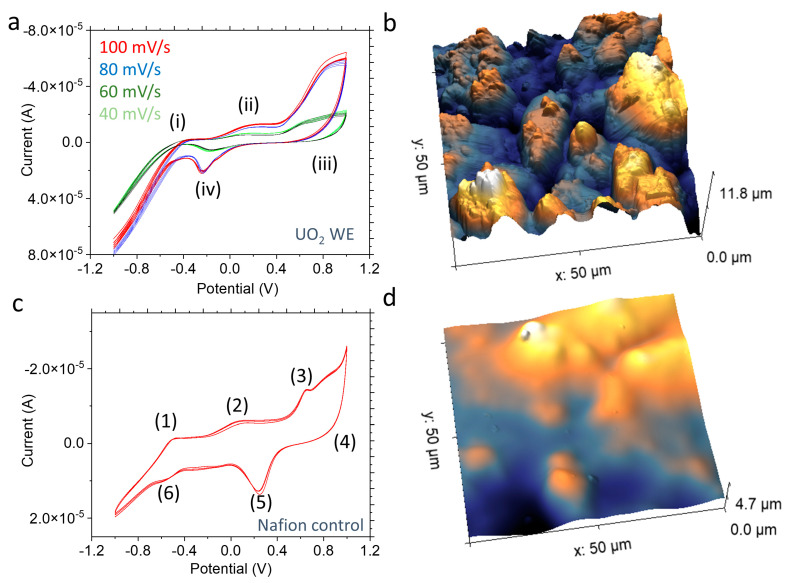
(**a**) The CV sweeping profile of the UO_2_ device B with different scanning rates (i.e., 40, 60, 80, 100 mV/s). Identified peaks are (i) −0.4 V, (ii) 0.13 V, (iii) 0.81 V, and (iv) −0.24 V; (**b**) AFM topography 3D image of the corroded UO_2_ WE surface; (**c**) CV scan profiles of the Nafion control device with a scan rate of 60 mV/s. Identified peaks are (1) −0.5 V, (2) 0.08 V, (3) 0.65 V, (4) 0.862 V, (5) 0.23 V, and (6) −0.53 V, and (**d**) AFM topography 3D image of the pristine UO_2_ WE surface.

This CV profile trend (Figure 3a) was reproduced in another UO_2_ WE device (device B) using the same test condition (100 mV/s scan rate). Similarly, the Nafion control’s CV profiles (Figure 3c) show similar peak potentials to those in Figure 2b, such as 0.65 V, 0.13 V, and −0.47 V, respectively. The CV profiles of device B are shown in Figure 3a, and they demonstrate device reproducibility at different scan rates. The peak potential profile pattern in Figure 2a and Figure 3a show similarities with a slight shift; the relevant matched peaks are as follows: (i) (−0.41V vs. −0.4), (ii) (0.11 vs. 0.13), (iii) (0.74 vs. 0.81), and (iv) (−0.23 vs. −0.24). Additional reproducibility results are shown in Figure S4. Figure 3c also shows the control device’s reproducibility with similar peak potential patterns. These results provide evidence of the DDE method that includes UO_2_ as an electrode. The potential peaks in the microfluidic devices do not exactly match those previously reported using bulk UO_2_ electrodes [30]. The slight potential peak shift between CV scans from Figure 2a and Figure 3a could be caused by the uncompensated ohmic drop effect. A high current density in microelectrodes can also distort the shape of the voltammogram and increase the overpotential [49,50]. Fabricated UO_2_ WEs are made in consistent dimensions; nevertheless, the improved, radiologically friendly fabrication tools can provide a more precise process. In addition, reference electrodes made of Pt wires were used in the E-cell, which differ from the bulk electrochemical setup, where a saturated calomel (Hg_2_Cl_2_) reference was used [20,24].

### 3.2. UO_2_ WE Surface Topographical Characterization

Figure 3b shows a relatively rough surface for the corroded UO_2_ electrode compared to the relatively smooth surface of the pristine UO_2_ WE in Figure 3d. This observation suggests that the Nafion membrane is suitable for attaching fine particles like UO_2_. That is, whether the particle is radioactive or not does not affect its electrochemical performance as expected. Figure S13a–c show AFM images of the smooth Nafion-only membrane surface for comparison [51]. Additional AFM results for the corroded UO_2_ electrode surface (Figure 3b) are shown in Figures S5 and S6. The CV results from the UO_2_ devices may have shown relatively more dominant Nafion because of the lack of access to the Nafion spin-coating option in the UO_2_-handling area. The Nafion layer on top of the as-prepared UO_2_ WE was thicker than those prepared using 1000 rpm spin-coated Nafion layer (Figures S1 and S2). This Nafion thickness could be reduced using the spin-coater during Nafion application [32]. Regardless, the thickness of the membrane does not stop the reactions. It only has the effect of reducing the signals slightly based on the analogue experiments [32]. Additionally, the differences between the UO_2_ device and the Nafion device demonstrate that the UO_2_ particles are indeed deposited.

### 3.3. XPS Analysis of the UO_2_ Electrode Surface

**Figure 4:**
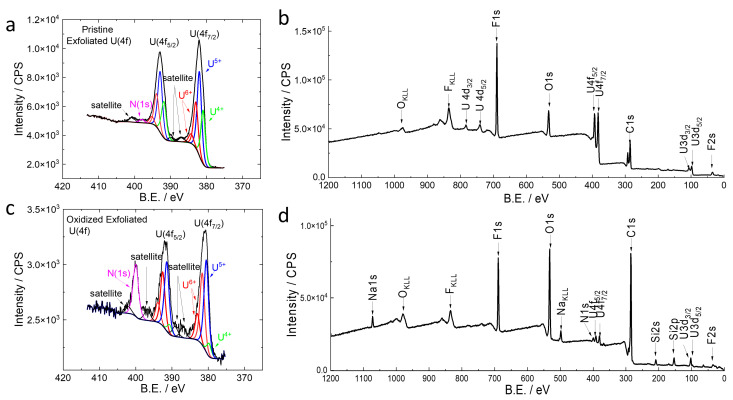
XPS spectra for as-synthesized exfoliated UO_2_ with (**a**) narrow-scan U(4f) and (**b**) wide-scan survey. The XPS spectra shown for the CV-scanned exfoliated UO_2_ with (**c**) narrow-scan U(4f) and (**d**) wide-scan survey. The narrow-scan U(4f) shows peak binding energies for the U(4f_7/2_), since the corresponding doublets in the U(4f_5/2_) region are fixed at a spacing of 10.9 eV and peak area ratio of 3:4 (U(4f_5/2_):U(4f(_7/2_)).

The pristine and corroded UO_2_ electrodes were exfoliated from the Si substrate (Figure S7) in an inert Argon (Ar) atmosphere to analyze the electrode surface adjacent to the gold conductive layer and the UO_2_ particles. The bottom side or the Au-Si substrate surface of each UO_2_ electrode was protected from further aerial oxidation, thus providing information on the true oxidation state of the UO_2_ before and after the electrically driven corrosion. The XPS narrow-scan region (Figure 4a) shows the near-surface uranium presence of the pristine UO_2_ electrode 29.2% of U(VI), which is likely related to UO_3_ due to exposure to air. Furthermore, 43.3% of U(V) is present, which could be attributed to U_3_O_8_. In the narrow scan, 27.5% of U(IV) is also observed as UO_2_ [43,52,53,54,55,56]. Aerial oxidation of the UO_2_ powder, along with exposure to water, likely explains the low UO_2_ presence in the original powder (Figure S8c). The presence of U_3_O_8_ is also expected because U_3_O_8_ could be gradually converted from UO_2_ at ambient temperatures. The comparison of XPS quantification results of exfoliated electrode surfaces shows an increase in U_3_O_8_ in the reacted UO_2_ WE (Table S2), suggesting that U(V) is a step due to interfacial oxidation and water interactions. The temperature effects that lead to U_3_O_8_ formation will be explored in future research.

In Figure 4c, the species distribution of the CV-scanned UO_2_ electrode is different from that of the pristine UO_2_ electrode. The calculated atomic percent values for the U(4f) core-level spectra (Table S2) show a clear sign of U(VI) increments from the oxidized UO_2_ electrode samples (oxidized exfoliated, and oxidized UO_2_). For example, the two major species are multivalent 45.2% of U(V), likely related to U_3_O_8_, and 40.9% of U(VI), likely related to UO_3_. The presence of U(IV) is noticeably decreased to 13.9%. Furthermore, substrate peaks are seen in the survey XPS spectrum (Figure 4d), which may indicate that the spectrum has signals from the thin Nafion membrane due to its redistribution on the electrode. This topographical change is verified by non-destructive AFM imaging (Figure 3b). Additional supporting results and related explanations [57] from the time-of-flight secondary ion mass spectrometry (ToF-SIMS) results are provided in Figure S11.

The characteristic satellite peaks observed from the pristine electrode [58,59,60] are not as prevalent after CV, which is likely due to the lower signal–noise ratio from the thinner film region of the electrode used for the analysis. Furthermore, after the corrosion of the UO_2_ electrode, the dominate species changes to U(VI), which is most likely attributed to UO_3_. Based on the XPS results, the most dominant species in the corroded UO_2_ electrode are multivalent U(V) and U(VI). Additionally, the Nafion membrane redistribution of the electrochemically reacted electrode surface was recently reported [32] using ToF-SIMS and AFM analyses of cerium oxide as an analogue of UO_2_. This phenomenon was also observed in UO_2_-corroded surfaces compared to pristine surfaces. Additional XPS narrow-scan results for the C(1s) region plots are shown in Figure S9. When comparing the prepared electrodes to the UO_2_ powder standard, some peak shifts occur, which are likely due to the preferential charging caused from the Nafion membrane (Figure S10).

Surface measurements, such as XPS and ToF-SIMS, provide quantification of the top few nm of the electrode material. The surface characterization results suggest that Nafion is not consumed. Nafion serves as a thin membrane to hold and protect the deposited UO_2_ particles, and it does not participate in the redox reactions. Nafion is an effective proton transporter used in proton-exchange membrane fuel cells. Water transport at the membrane and the electrode surface has significant impact on the electrochemical performance. Four types of water inside the membrane have been identified, including protonated water, water H-bonded to other water molecules, water H-bonded to functional groups, and non-H-bonded water [61,62]. The ex situ measurements cannot classify the type of water in the UO_2_ particle microenvironment. Raman spectral comparison results are also used (Figure S12). The broad peak in the Raman spectra above 3000 cm^−1^ in the corroded electrode can be attributed to the OH vibrations [63,64]. This finding indicates that interfacial water uptake could occur during the electrode electrochemical process. In addition, OH vibrations are not observed in the bulk electrodes after water treatment, confirming that the water molecules are only absorbed during the electrochemical reaction [63,64].

### 3.4. Interfacial Water and Possible UO_2_ Reaction Pathways

It is worth noting that the oxidation process of spent fuel could be driven by various oxidants, including water radiolysis, pH, temperature, groundwater composition, the formation of corrosion products, and deposition [3,19,20,21,22,23,24,25,30]. In general, groundwater at the depth of a repository is expected to contain little oxygen. However, radiolysis will produce oxidants upon groundwater intrusion into failed spent fuel canisters, followed by the ionization of U(IV). It is likely that a small fraction of U(IV) ions are oxidized to form the U(V) and/or U(VI) valence states, leading to the creation of holes in the narrow occupied U5f sub-band [3]. These holes can migrate by a small polaron-hopping process with a low activation energy and confer a moderate conductivity to the oxide [3,24]. To date, most spent fuel corrosion studies cover the oxidation process from U(IV) to U (VI) state by H_2_O_2_ and pH change [8,22,24] or through H_2_O and hydrothermal conversion [65,66].

**Figure 5:**
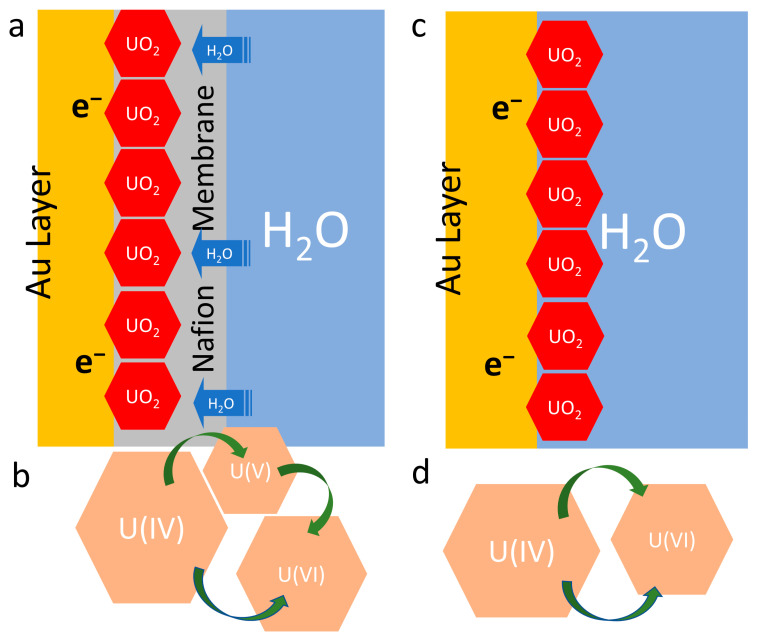
Schematics showing the UO_2_ oxidation process of (**a**) the UO_2_ electrode with a Nafion layer which allows for limited H_2_O exposure to the UO_2_ particle layer and (**b**) its corresponding reaction route diagram using microscale cells compared to (**c**) the fully H_2_O-exposed UO_2_ electrode from previous works [24], and (**d**) its corresponding reaction route diagram.

In the natural environment, U(V) exists in the mixed-valence mineral wyartite and in a U oxide state such as U_3_O_8_. However, there are limited experimental studies about the conversion from U(IV) to U(V) [67,68]. Among the reactions of spent fuel, the most common source of U(V) is from U_3_O_8_ that is transformed from UO_2_. Several U_3_O_8_ studies reported that there were mixtures of U(V) and U(VI) [26,68], and that U(V) was not the dominant species obtained from such reactions. The effect of temperature was not investigated. The observation of U(V) is a consequence of the inclusion of interstitial oxygen as the oxidant via either chemical or electrochemical processes. U(V) is present in a thin surface layer and detectable by XPS under the conditions of this study. Figure 5 depicts the proposed mechanism for the observed U(IV) to U(V)/U(VI) reaction routes with the previously presented U(IV) to U(VI) reactions. It is postulated that the Nafion membrane’s selective hydro-semi-permeability and the radiolysis of surrounding water play a key role in the U(IV) to U(V)/U(VI) route (Figure 4a,b).

To support the major U(IV) to U(VI) route interpretation, the surface tension of the UO_2_-containing electrode surfaces relevant to the electrolyte used in corrosion conditions was determined to provide the physical properties. The surface tension measurements (see Table S4) show that the Nafion coating lowers surface tension, indicating that the surface becomes more hydrophobic. However, after the electrochemical corrosion, the electrode surface has higher surface tension compared to the pristine one (e.g., 24.25 mN/m vs. 15.76 mN/m), suggesting that the redox reactions make the corroded electrode surface more hydrophilic (Table S4b). This finding supports the hypothesis of interfacial water depicted in Figure 5d. Namely, water molecules could enter between UO_2_ particles after Nafion is redistributed during CV sweeping. Consequently, UO_2_ particles will have more surrounding water molecules after electrochemical corrosion. It is postulated that UO_2_ particles will partake in relatively limited oxidation reactions compared to the fully exposed situation. In the latter, the macro-sized electrode is completely soaked in an aqueous electrolyte [69,70,71]. The minor U(IV) to U(V) route contributing to the slight increase (Table S2) after the CV scan also can be explained by an electron transfer reduction and thermal oxidation due to microscale surface-level temperature elevations [67,68,72]. This requires further study in future efforts.

As previously reported, Nafion has a very selective, semi-permeability to water, which is dependent on temperature and the volume fraction of water in the membrane [32,73,74]. The hydraulic permeability of Nafion is small relative to the diffusive transport of water in some cases. This may explain the appearance of U(V)/U(VI) as the product of the corroded UO_2_ electrode. In this, the UO_2_ particles will be exposed to surrounding water molecules in the bulk phase experiments performed in earlier reports [19,21,24,25]. The water semi-permeable Nafion layer on top of the UO_2_ particles may cause the U(IV) to U(V)/U(VI) transformation, which is most likely attributed to the interfacial water. Assuming that there is no major physical failure in the repository during long-term spent fuel storage, a double-walled canister corrosion failure is one of the major concerns. In the latter, groundwater intrusion would be a main concern [2,6,75]. This U(IV) to U(V)/U(VI) transformation scenario is relevant when cracks in the failed canister gradually introduce groundwater into the stored spent fuel. Thus, the results and the device configuration developed in this work can offer a new venue to study UO_2_ under relevant storage conditions. For example, altering the temperature for Nafion’s water permeability shift may provide a similar environment to the failed spent fuel canister. This aspect could be important to the Fuel Matrix Dissolution Model (FMDM) development [8], since the water-lean condition has not been thoroughly explored. Considering the interfacial water and the UO_2_ interaction scenario could potentially expand the FMDM and increase the model predictability. It is worth noting that Nafion’s selective hydrophobicity provided a more relevant physical model of water and spent fuel during the limited exposure to water scenario than any other material-treated surfaces, such as a graphite-treated surface, because graphite surfaces are intrinsically mildly hydrophilic [42]. If the surface is treated with finer graphite particles, such as carbon black powder, it would be hydrophilic, similar to a surface that is fully exposed to water.

Since the SALVI E-cell is compatible with multiple analytical platforms, adding UO_2_ particles to the E-cell permits a multimodal and in operando analysis of spent fuel materials in the future. Using microscale devices to study the spent fuel also would allow for other types of particles that could be difficult to acquire and handle at the macroscale. For example, synfuels or noble metal particles could now be incorporated into the devices in a controlled manner in a portable platform instead of using the ex situ approach presented in previous works [18,24,30].

### 3.5. Reducing and Separating the Radiation Effect Using Miniturization

The radiation measurement of the fabricated UO_2_ WE chips was based on α particle and β-γ particle counts, respectively. The α-particle count was considered negligible (>100 cpm). The β/γ-particle count measurements of four WE chips are presented in Table 1. These results show relatively high UO_2_ mass loadings in two chips, namely no. 1 and no. 2 samples. Their corresponding β-γ particle counts were higher than those of the no. 3 and no. 4 electrode chips, which were recovered from devices A and B. This result indicates that reducing the amount of UO_2_ particles on the electrode surface will meaningfully decrease the β-γ radiation contribution from the spent fuel particles (or similar substances). Using a lower UO_2_ mass loading could lead to negligible radiation counts in the future. This is an intrinsic strength that the SALVI E-cell provides.

## 4. Conclusions

UO_2_ spent fuel particles are used as electrodes in miniaturized electrochemical cells. A layer of thin Nafion membrane was used to protect the UO_2_ particles, with verified reproducibility and electrochemical performance. When UO_2_ particle analysis is not logistically feasible, CeO_2_ is used as a reliable analog to assist the device and method development and to reduce radiological waste during feasibility testing. The Nafion-based method can provide more precise UO_2_ mass loadings, stability, and reproducibility in terms of electrochemical performance for studies of spent fuel corrosion. Furthermore, the as-prepared pristine and the electrochemically redox-processed WE surfaces were characterized using AFM, suggesting Nafion redistribution as a result of electrochemical reactions. Also, the quantitative uranium presence ratios from XPS show differences in the U(IV), U(V), and U(VI) distribution between the pristine and corroded UO_2_ electrode surface. The new results suggest that a U(IV) to U(V) route exists in addition to the known U(IV) to U(VI) pathway. We postulate that the interfacial water plays a key role in the U(IV) to U(V) reaction step due to the selective nature of the semi-permeability of the Nafion membrane of water. This “water-lean” configuration could mimic the limited water exposure of the spent fuel in a failed storage canister, presenting a scenario that is not compatible with a study using bulk electrodes.

This work provides a valuable new electrochemical tool for studying spent fuel materials and understanding their corrosion potential. This new solution will offer a flexible platform to introduce SIMFUEL, noble metal particles, or controlled dopants in electrode preparation and simultaneously separate the α- from β/γ effects. This is because α particles have a limited ability to penetrate other materials; therefore, one can use small devices to separate the α-particle effect from β and γ. It is anticipated that more systematic research will be performed using microscale electrochemical cells to study the spent fuel corrosion chemistry at the material interface.

## Figures and Tables

**Table 1:** The β–γ particle count results from four UO_2_ electrode chips.

No	UO_2_ Electrode Description	β–γ Particle Count (Count per min, cpm)	UO_2_ Particle Mass (mg)
1	high loading of UO_2_ WE #1	955	33
2	high loading of UO_2_ WE #2	637	33
3	lower loading of UO_2_ WE #A	299	1.86
4	lower loading of UO_2_ WE #B	438	3.72

## Data Availability

The data presented in this study are available on request from the corresponding author.

## Supplementary Materials

The following supporting information can be downloaded at: https://www.mdpi.com/article/10.3390/mi14091727/s1, Figure S1: Photos of Nafion membranes (a) 5 wt.% Nafion spun at 500 rpm, (b) 20 wt.% Nafion spun at 1000 rpm, and (c) 20 wt.% Nafion spun at 500 rpm, on top of CeO_2_ particles deposited on clean Si chips. (d) The profilometer measurement of CeO_2_ particles covered with a 20 wt.% Nafion spun at 500 rpm. All were on the 2 × 2 mm^2^ Si chips; Figure S2: Nafion membrane thickness measurements using a profilometer, (a) 1000 rpm, (b) 500 rpm, and (c) drop-applied; Figure S3: Optical images of the 2 mm × 2 mm CeO_2_ electrode area of (a) the low and (b) high-mass-loading electrode and (c) the profilometer measurement results of the electrode; Figure S4: CV reproducibility results of E-cell using CeO_2_ particles as an analogue of UO_2_. Scan rate of (a)10 mV/s, (b) 20 mV/s, (c) 40 mV/s, (d) 60 mV/s, (e) 80 mV/s, and (f) 100 mV/s; Figure S5: (a) Optical image of the UO_2_ WE device after CV, (b) corresponding AFM topography, and (c) cursor plot from the indicated line in (b). (d) Optical image of the pristine UO_2_ WE device, (e) corresponding AFM topography, and (f) cursor plot from the indicated line in (e); Figure S6: (a–d) Consecutive AFM topography of UO_2_ WE device After CV where (d) is from the white marked region from (a), and (b) and are from the white marked region from (d), (c) 3D topography, (e) corresponding amplitude image of (e), and (f) cursor plot of the marked line from (b); Figure S7: Photos of the exfoliated (a) oxidized and (b) pristine UO_2_ WE surface. The red dashed squares indicate the XPS analysis areas; Figure S8: XPS spectral results for the narrow-scan U(4f) region of (a) pristine UO_2_ WE, (b) oxidized UO_2_ WE, (c) and pristine UO_2_ powder surfaces along with the corresponding wide-scan survey plots. The U(4f7/2) peaks are identified with their respective binding energies with their doublet U(4f5/2) fixed at a difference of 10.9 eV due to spin–orbit coupling and a peak area ratio of 3:4 with the corresponding U(4f7/2); Figure S9: XPS narrow-scan results for the C(1s) region for (a) powder UO_2_ reference, (b) pristine and (c) oxidized exfoliated along with (d) pristine and (e) oxidized UO_2_; Figure S10: XPS spectral comparison of the U(4f) region for the electrodes with powder UO_2_ reference; Figure S11: Normalized ToF-SIMS spectral comparison of the oxidized (a) and pristine (b) electrode surfaces; Figure S12: Raman spectral comparison of (a) freshly harvested corroded electrode from a SALVI E-cell device, (b) pristine electrode, (c) corroded electrode with water treatment, and (d) pristine electrode with water treatment; Figure S13: AFM height and amplitude images of ~35 nm thick films of Nafion. (a–c) various conditions of Nafion and calix-2 with IECs 2.8 (d–f); 3.9 (g–i); and 5.8 (j–l). The scale bars are shown within the images. by Chatterjee el. Al. JACS Au 2022, licensed under CC BY-NC-ND 4.0 (https://pubs.acs.org/doi/10.1021/jacsau.2c00143?fig=fig4&ref=pdf accessed on 1 July 2023). Table S1: Electrode sample descriptions for the AFM and XPS analysis; Table S2: Calculated atomic percent values for the U(4f) core level spectra; Table S3: Quantification of the Nafion membrane using the F(1s) peak from the survey spectrum and C-F peak from the C(1s) with peak areas normalized with the Kratos library relative sensitivity factor (RSF) for C(1s). Relative C-F contribution for the C(1s) narrow scan is also reported using atomic percentages (at%); Table S4a: Sample descriptions of the surface tension measurements; Table S4b: Summary of surface tension measurements of the electrodes.
