# Supplementary material for: Microscale Electrochemical Corrosion of Uranium Oxide Particles
Source: Micromachines (Basel). 2023 Sep 1;14(9):1727. doi: 10.3390/mi14091727 (PMC10537459; doi:10.3390/mi14091727)
Supplement: Supplementary file 1 [file micromachines-14-01727-s001.zip › micromachines-2517794-supplementary.pdf]

micromachines

MDPI

Supporting Information

Studying Uranium Oxide Corrosion at the Electrode Surface Using Microfluidics

Jiyoung Son,[a] *Shawn L. Riechers,*[a] *and Xiao-Ying Yu,*[b, *]

[a] Energy and Environment Directorate, Pacific Northwest National Laboratory, Richland, WA 99354
[b] Materials Science and Technology Division, Oak Ridge National Laboratory, Oak Ridge, TN 37830

E-mail: yuxiaoying@ornl.gov; Tel: 865-574-4628
Dr. Xiao-Ying Yu
Materials Science and Technology Division, Oak Ridge National Laboratory, Oak Ridge, TN 37830-6136

Contents

Supporting Experimental Details 4

Nafion-UO_2 WE fabrication step development 4

Fabrication of SALVI E-cell device 4

XPS analysis setting and sample preparation 4

Surface Tension 5

Supplemental Tables 6

Table S1. Electrode sample descriptions for the AFM and XPS analysis. 6

Table S2. Calculated atomic percent values for the U(4f) core level spectra. 7

Table S3. Quantification of the Nafion membrane using the F(1s) peak from the survey spectrum and C-F peak from the C(1s) with peak areas normalized with the Kratos library relative sensitivity factor (RSF) for C(1s). Relative C-F contribution for the C(1s) narrow scan is also reported using atomic percentages (at%). 8

Table S4a. Sample descriptions of the surface tension measurements. 9

Table S4b. Summary of surface tension measurements of the electrodes. 9

Supplemental Figures 10

Figure S1. Photos of Nafion membranes a) 5 wt% Nafion spun at 500 rpm, b) 20 wt% Nafion spun at 1000 rpm, and c) 20 wt % Nafion spun at 500 rpm, on top of CeO_2 particles deposited on clean Si chips. d) The profilometer measurement of CeO_2 particles covered with a 20 wt% Nafion spun at 500 rpm. All were on the 2×2 mm^2 Si chips. 10

Figure S2. Nafion membrane thickness measurements using a profilometer, (a) 1000 rpm, (b) 500 rpm, and (c) drop applied. 11

Figure S3. Optical images of the 2 mm x 2 mm CeO_2 electrode area of (a) the low and (b) high mass loading electrode and (c) the profilometer measurement results of the electrode. 12

Figure S4. CV reproducibility results of E-cell using CeO_2 particles as an analogue of UO_2. Scan rate of a)10 mV/s, b)20 mV/s, c) 40 mV/s, d) 60 mV/s, e) 80 mV/s, and f) 100 mV/s. .. 13

Figure S5. (a) Optical image of the UO_2 WE device after CV, (b) corresponding AFM Topography, and (c) cursor plot from the indicated line in (b). (d) Optical image of the pristine UO_2 WE device, (e) corresponding AFM Topography, and (f) cursor plot from the indicated line in (e). 14

Figure S6. (a-d) Consecutive AFM Topography of UO_2 WE device After CV where (d) is from the white marked region from (a), and (b) and are from the white marked region from (d), (c) 3D Topography, (e) corresponding amplitude image of (e), and (f) cursor plot of the marked line from (b). 15

Figure S7. Photos of the exfoliated (a) oxidized and (b) pristine UO_2 WE surface. The red dashed squares indicate the XPS analysis areas. 16

Figure S8. XPS spectral results for the narrow scan U(4f) region of (a) pristine UO_2 WE, (b) oxidized UO_2 WE, (c) and pristine UO_2 powder surfaces along with the corresponding wide scan survey plots. The U($4f_{7/2}$) peaks are identified with their respective binding energies with

their doublet U($4f_{5/2}$) fixed at a difference of 10.9 eV due to spin-orbit coupling and a peak area ratio of 3:4 with the corresponding U($4f_{7/2}$)....... 17

Figure S9. XPS narrow scan results for the C(1s) region for (a) powder UO_2 reference, (b) pristine and (c) oxidized exfoliated along with (d) pristine and (e) oxidized UO_2....... 18

Figure S10. XPS spectral comparison of the U(4f) region for the electrodes with Powder UO_2 reference....... 19

Figure S11. Normalized ToF-SIMS spectral comparison of the oxidized (a) and pristine (b) electrode surfaces....... 20

Figure S12. Raman spectral comparison of (a) freshly harvested corroded electrode from a SALVI E-cell device, (b) pristine electrode, (c) corroded electrode with water treatment, and (d) pristine electrode with water treatment....... 21

Figure S13. AFM height and amplitude images of ~35nm thick films of Nafion. (a-c) various conditions of Nafion and calix-2 with IECs 2.8 (d–f); 3.9 (g–i); and 5.8 (j–l). The scale bars are shown within the images.[77] by Chatterjee el. Al. JACS Au 2022, licensed under CC BY-NC-ND 4.0 (https://pubs.acs.org/doi/10.1021/jacsau.2c00143?fig=fig4&ref=pdf)....... 22

References....... 23

Additional experiment details, figures and tables are provided to support the content in the main text.

Supporting Experimental Details

Nafion-UO_2 WE fabrication step development

We suspended UO_2 particles (186 mg) into 0.1 mL of DI water. The working electrode (WE) has about few mg of UO_2 particles within the 2 x 2 mm^2 Kapton tape (3M, Saint Paul, MN, USA) masked Au-WE surface by pipetting few droplets of suspended UO_2 solution (**Table 1**). The Si chips with UO_2 particles were dried in room temperature. Thin Nafion layer was fabricated on top of the deposited particles by pipetting 10 µL of the Nafion solution. After applying the Nafion solution, the UO_2 WE Si chip had been dried in room temperature for a day. During the UO_2 WE fabrication, we also altered the amount of UO_2 particles on the WE to determine the minimum mass of particles needed for negligible radiation by altering the number of droplets. We surveyed each fabricated UO_2 WE chip for α-particles and β-γ particles counts by using a digital radiation scanner (Model 2929, Ludlum). The fabricated UO_2 WE chips were used to make SALVI E-cells. The E-cell is a three-electrode system. Two platinum (Pt) wires are used as the reference (RE) and the counter electrode (CE) [33, 2]. It is worth noting that all UO_2 handling activities were performed under an approved radiological work protocol in the proper laboratory space at PNNL.

Fabrication of SALVI E-cell device

Fabrication of SALVI E-cell is based on previous works [31,33,35,74]. In brief, the fabricated WE was bond to-gether with an electrochemical cell chamber made of the polydimethylsiloxane (PDMS) block by plasma surface activation. An electrochemical chamber is ellipsoid shaped with a width 7 mm long, 3 mm wide, and 1.5 mm deep. Four holes were punched through the PDMS at each end of the microchannel for fluid injection tubing and CE/RE using a biopsy punch (WPI, Sarasota, FL, USA). Metal fluidic tubing was made from 90 bended metal tubing to connect the microchannel with the PTFE tubes (Sigma, Aldrich, St. Louis, MI, USA). A piece of silver wire (0.25 mm thick, 5 cm long) (Alfa Aesar, Ward Hill, MA, USA) is attached on Au substrate of WE using silver epoxy (CircuitWorks, Chemtronics, Kennesaw, GA, USA). Two platinum wires (0.25 mm thick, 5cm long) (Alfa Aesar, Ward Hill, MA, USA) were inserted into the punched hole of PDMS cell chamber. The ends of these two PTFE tubes were connected by a PEEK union and fittings (Upchurch, Oak Harbor, WA, USA).

XPS analysis setting and sample preparation

X-ray Photoelectron Spectroscopy (XPS) was performed on a Kratos AXIS Ultra DLD system using a monochromatic Al-Ka source (hv = 1486.7 eV) operating at an analysis chamber pressure of $< 2 \times 10^{-9}$ Torr. The working electrodes were inserted into an anoxic glove box filled with argon that is connected to the fast entry-port of the XPS instrument. For the samples labeled as 'exfoliated', a double-sided 3M copper tape was used to press on to the UO_2/Nafion WE and then slowly peel the tape off. This enabled us to probe the material that was closest to the electrode. The Si chip with the working electrode (or with the exfoliated material) was then transferred into the instrument via the load-lock and XPS analysis was performed. During analysis, any surface charging was minimized by using a low-energy electron flood gun. Survey spectra was acquired at a pass energy (PE) of 160 eV and a step size of 1 eV, while high-resolution data was acquired at a PE of

40 eV with a step size of 0.1 eV. For reference, the Au $4f_{7/2}$ feature of a sputter-cleaned Au foil yielded a full width at half maximum (FWHM) of 1.9 eV at a PE of 160 eV, while a PE of 40 eV gives a FWHM of 0.8 eV. The acquired data was processed using Casa XPS software and was charge referenced to adventitious C 1s (C-C/C-H component) at 285 eV. The U(4f) narrow scan spectra were fit using Gaussian-Lorentzian type curves with a Shirley background subtraction [55].

Surface Tension

The surface tension measurements of the **CeO_2 WE** and reference samples were performed using a KRUESS K12 Tensiometer (KRUESS, Hamburg, Germany). Each sample was prepared to represent the CeO_2 Nafion WE (Samples 5, 6) and its reference surfaces namely, the clean Si wafer chip (Sample 1), Au-Ti coated Si chip (Sample 2), CeO_2 deposited Au-Ti coated Si chip (Sample 3), and Nafion coated on Au-Ti layer on a clean Si chip (Sample 4). The surface tension change was compared between the pristine (Sample 5) and CV scanned CeO_2 (Sample 6) WE surface. The sample size was 1 x 1 cm^2 in the x- and y- laterally and 0.5 mm thick. 0.1 M of sodium perchlorate ($NaClO_4$) was used as the wetting liquid during measurements. The temperature of the liquid was 24.2°C. Each sample measurement was repeated three times to generate average values and standard deviations. The surface tension results are summarized in **Table S3**.

Supplemental Tables

Table S1. Electrode sample descriptions for the AFM and XPS analysis.

No.	Sample Name	Sample Description
1	Oxidized UO_2 WE Exfoliated	Exfoliated UO_2 WE and Au layer from Oxidized UO_2 WE chip after CV scan
2	Oxidized UO_2 WE	Oxidized UO_2 WE chip after CV scan
3	Pristine UO_2 WE Exfoliated	Exfoliated UO_2 WE with Au layer from pristine UO_2 WE chip
4	Pristine UO_2 WE	Pristine UO_2 WE chip before CV scan
5	Powder UO_2	Source UO_2 powder used for building UO_2 WE

Table S2. Calculated atomic percent values for the U(4f) core level spectra.

Electrode	U^{4+} At %	U^{5+} At %	U^{6+} At%
Powder UO_2 Reference	32.4%	23.6%	43.9%
Pristine Exfoliated	27.5%	43.3%	29.2%
Oxidized Exfoliated	13.9%	45.2%	40.9%
Pristine UO_2	41.0%	34.6%	24.3%
Oxidized UO_2	34.0%	27.6%	38.3%

The values shown in **Table S2** are the calculated at% for the deconvoluted U(4f) core level spectra. The powder UO_2 reference shows oxidation to U(VI):UO_3 and (V):U_3O_8. The pristine exfoliated fabricated electrode shows an increase in U^{6+} after electrochemical cycling whereas the pristine UO_2 electrode shows a slight increase in U^{5+} at the surface. Both electrodes show a decrease in in UO_2, which implies electrochemical oxidation is taking place.

Table S3. Quantification of the Nafion membrane using the F(1s) peak from the survey spectrum and C-F peak from the C(1s) with peak areas normalized with the Kratos library relative sensitivity factor (RSF) for C(1s). Relative C-F contribution for the C(1s) narrow scan is also reported using atomic percentages (at%).

Electrode	F(1s) Normalized Peak Area	C-F C(1s) Normalized Peak Area	C-F at % relative to entire C(1s) region
Powder UO_2 Reference	N/A	N/A	N/A
Pristine Exfoliated	29180.9	22460.0	28.7%
Oxidized Exfoliated	15349.3	10842.1	7.5%
Pristine UO_2	51355.8	47446.8	40.8%
Oxidized UO_2	18969.4	17738.5	28.0%

Table S3 reports the values used to quantify the Nafion peak on the electrode using two different approaches: (1) using the F(1s) peak area of the survey spectrum which is normalized using the RSF and (2) using the C-F component of the C(1s) narrow scan region normalized using the RSF. The relative atomic percent of the C-F compared to the entire C(1s) region is also reported. After oxidation, the F(1s) normalized peak area decreases to almost half of the pristine amount, which may indicate the measurement was taken over a thinner region of the electrode or that some of the Nafion membrane is redistributed during electrochemical cycling. The same trend is also observed for the C-F component of the C(1s) region along with relative atomic percentages of C-F to the entire C(1s) region.

Table S4a. Sample descriptions of the surface tension measurements.

No	Sample Descriptions
1	The plane Si chip
2	The Au-Ti layer on top of the clean Si surface
3	The surface with the CeO_2 powder deposited on the Au-Ti layer
4	The Nafion layer on top of Si wafer
5	The Nafion layer on top of CeO_2 powders as a pristine WE
6	The Nafion layer on top of CeO_2 powders after CV scans as the corroded WE

Table S4b. Summary of surface tension measurements of the electrodes.

Sample No.	1	2	3	4	5	6
Average Surface Tension (mN/m)	45.3	44.33	42.18	37.06	15.76	24.25
Standard Deviation of Surface Tension(mN/m)	0.39	0.15	7.27	1.38	1.22	3.81
Calculated Contact Angle (degree)	89.98	89.98	89.97	89.98	89.94	89.96

There is a clear change of surface tension between the Nafion applied surface and that without Nafion. Surface tension of the non-Nafion surface is higher than that with Nafion covering by ~70% (i.e., Sample 1 vs. Sample 5). This means Nafion makes the surface more hydrophobic compared to other reference samples (i.e., Samples 1 – 3). The hydrophobicity also changes because of the electrochemically driven Nafion reformation. The surface tension of the electrode surface post CV scanning (i.e., Sample 6) increases compared to the pristine surface (i.e., Sample 5). This result indicates that CV scans drive Nafion reformation and make the surface more hydrophilic compared to the unused Nafion surface (e.g., Sample 5).

Supplemental Figures

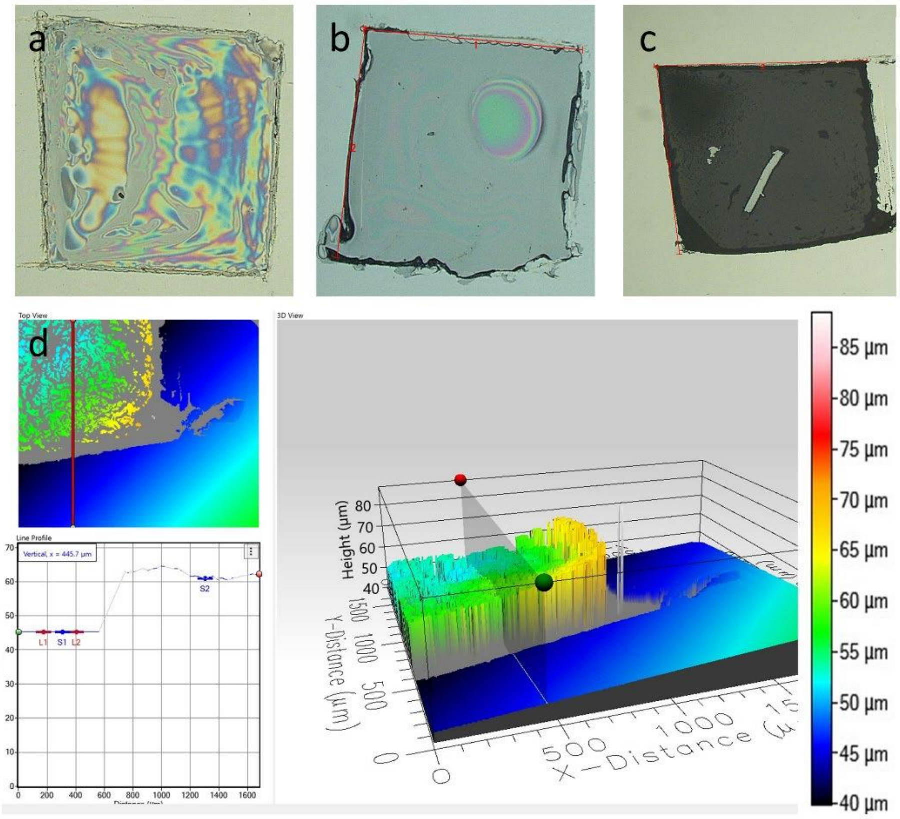

Figure S1. Photos of Nafion membranes a) 5 wt% Nafion spun at 500 rpm, b) 20 wt% Nafion spun at 1000 rpm, and c) 20 wt % Nafion spun at 500 rpm, on top of CeO_2 particles deposited on clean Si chips. d) The profilometer measurement of CeO_2 particles covered with a 20 wt% Nafion spun at 500 rpm. All were on the 2×2 mm^2 Si chips.

A thin layer of Nafion could form a solid membrane, when they were applied orderly on the Si surface as shown in **Fig. S1**. Curing in oven is necessary for the Nafion membrane to form [31]. More fabrication and measurement details of the CeO_2 particle attached electrodes were described in the experimental supplemental section and a recent paper. [31]

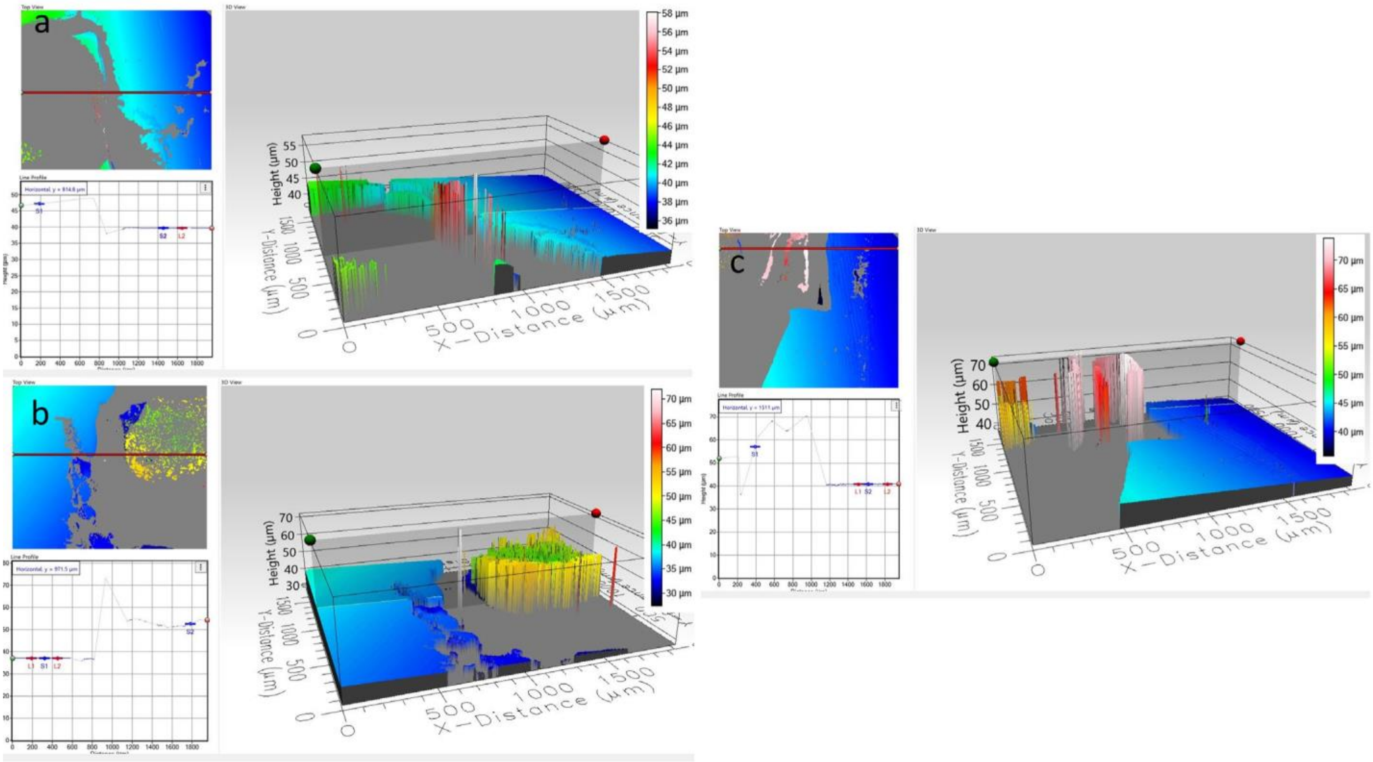

Figure S2. Nafion membrane thickness measurements using a profilometer, (a) 1000 rpm, (b) 500 rpm, and (c) drop applied.

Using CeO_2 particles as an analogue of UO_2, the Nafion membrane formed on top of particles were measured using a profilometer. **Figure S2** depicts measurement results using with 5 wt% Nafion solution with three different spinning rates. The results show that faster spinning rate helps form an even and thin layer. The total WE thickness ranges of (1000 rpm, 500 rpm, and drop) are ~6, ~12, and ~14 µm respectively, based on measurements using a profilometer. More detailed membrane thickness conditions can be found in our previous report. [31]

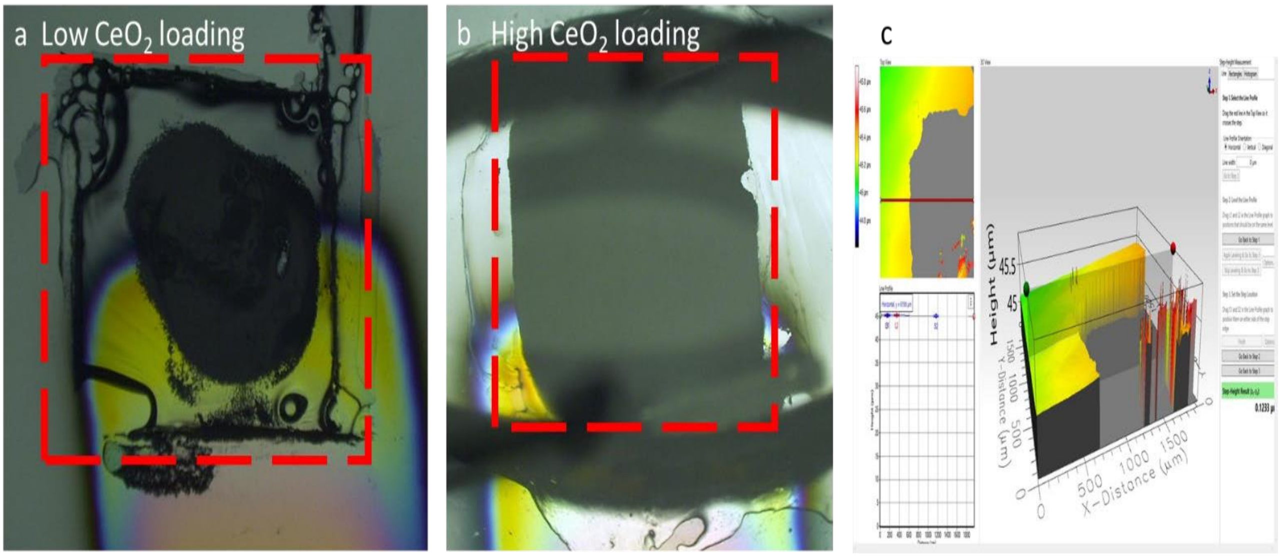

Figure S3. Optical images of the 2 mm x 2 mm CeO_2 electrode area of (a) the low and (b) high mass loading electrode and (c) the profilometer measurement results of the electrode.

Using a high loading of CeO_2 of 6 mg the surface profilometer thickness measurement shows that the average thickness is 0.15 μm over an area of ~2 × 2 mm^2. When using a lower CeO_2 loading of 0.06 mg, the thickness is less. In each case, 24 μL of the liquid mixture was taken from of a stock by pipetting to the gold conductive layer.

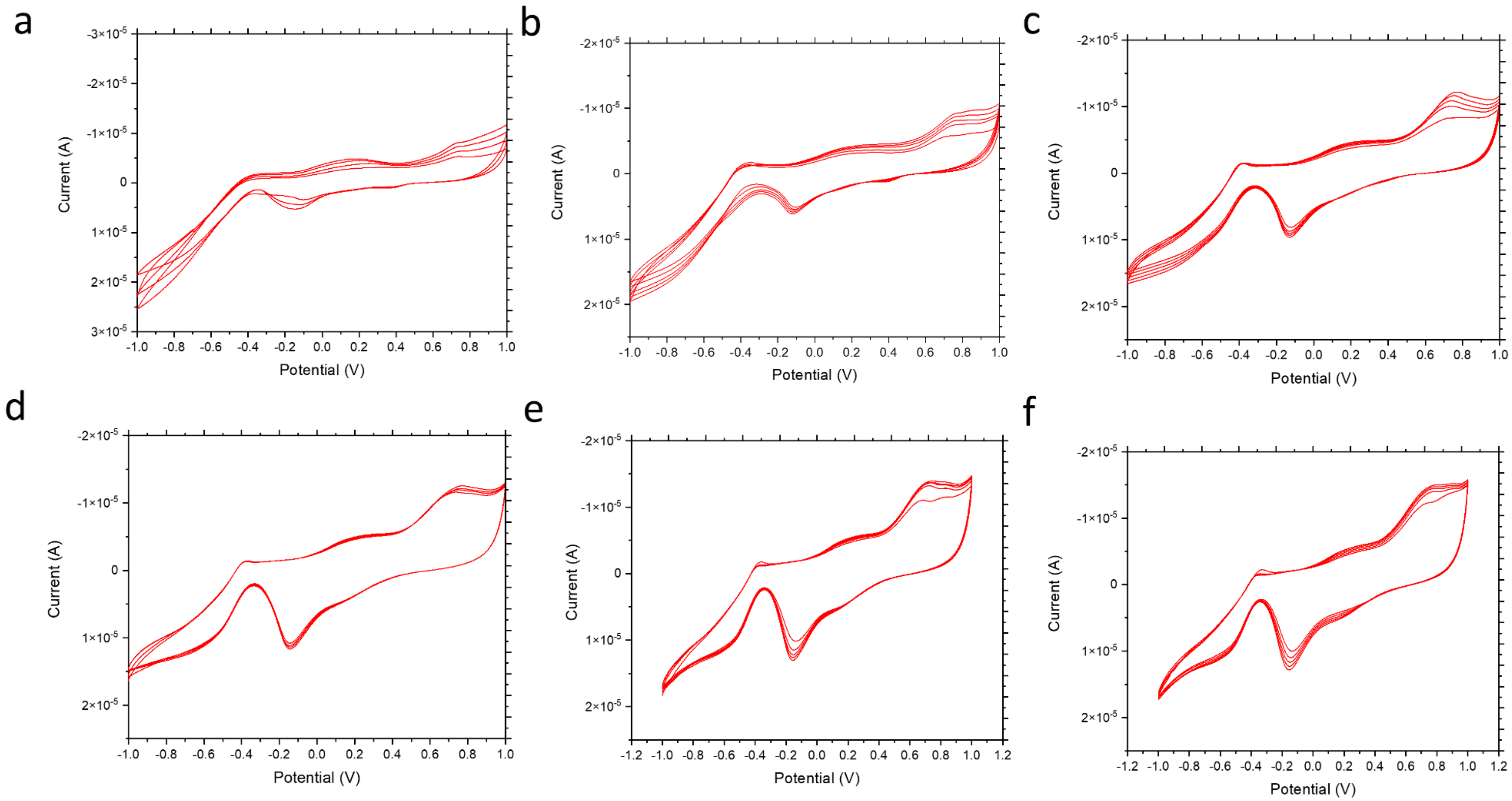

Figure S4. CV reproducibility results of E-cell using CeO_2 particles as an analogue of UO_2. Scan rate of a)10 mV/s, b)20 mV/s, c) 40 mV/s, d) 60 mV/s, e) 80 mV/s, and f) 100 mV/s.

Before applying the WE fabrication method to UO_2, CeO_2 particles were used as an analogue to improve conditions and reach an optimized procedure [31]. The CeO_2 particles were deposited onto the gold conductive layer. Then a thin Nafion layer was formed by spinning 5 wt% Nafion solution at 1000 rpm.

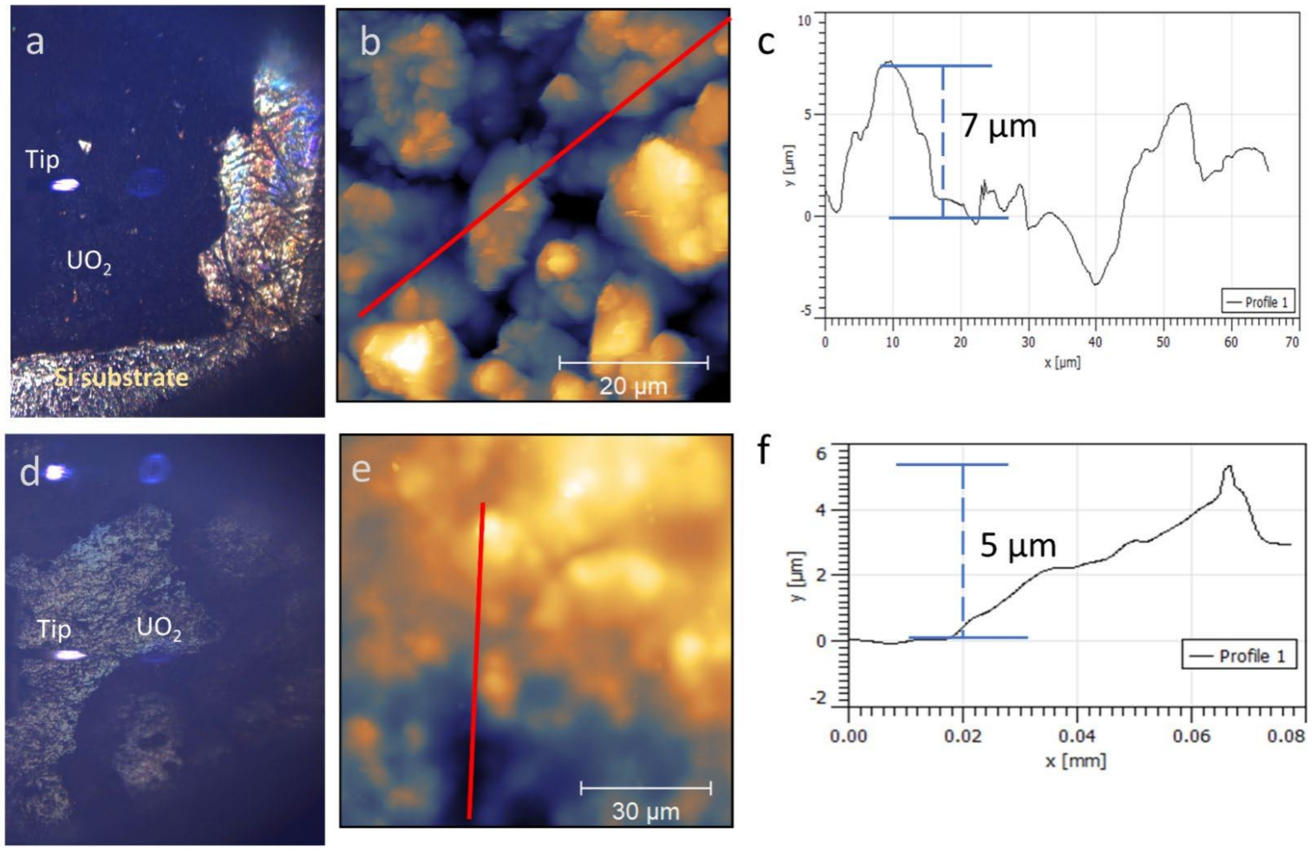

Figure S5. (a) Optical image of the UO_2 WE device after CV, (b) corresponding AFM Topography, and (c) cursor plot from the indicated line in (b). (d) Optical image of the pristine UO_2 WE device, (e) corresponding AFM Topography, and (f) cursor plot from the indicated line in (e).

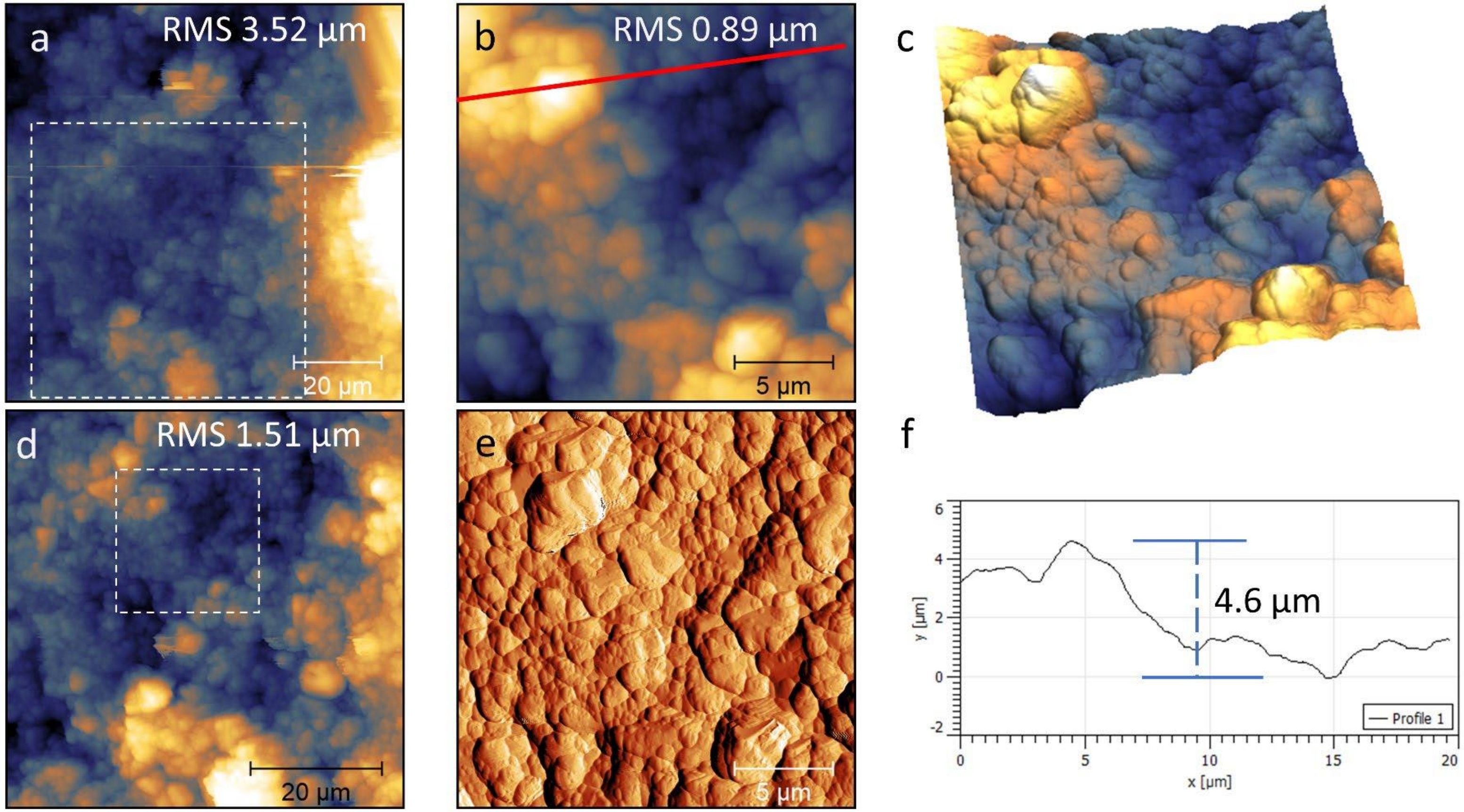

Figure S6. (a-d) Consecutive AFM Topography of UO_2 WE device After CV where (d) is from the white marked region from (a), and (b) and are from the white marked region from (d), (c) 3D Topography, (e) corresponding amplitude image of (e), and (f) cursor plot of the marked line from (b).

micromachines

MDPI

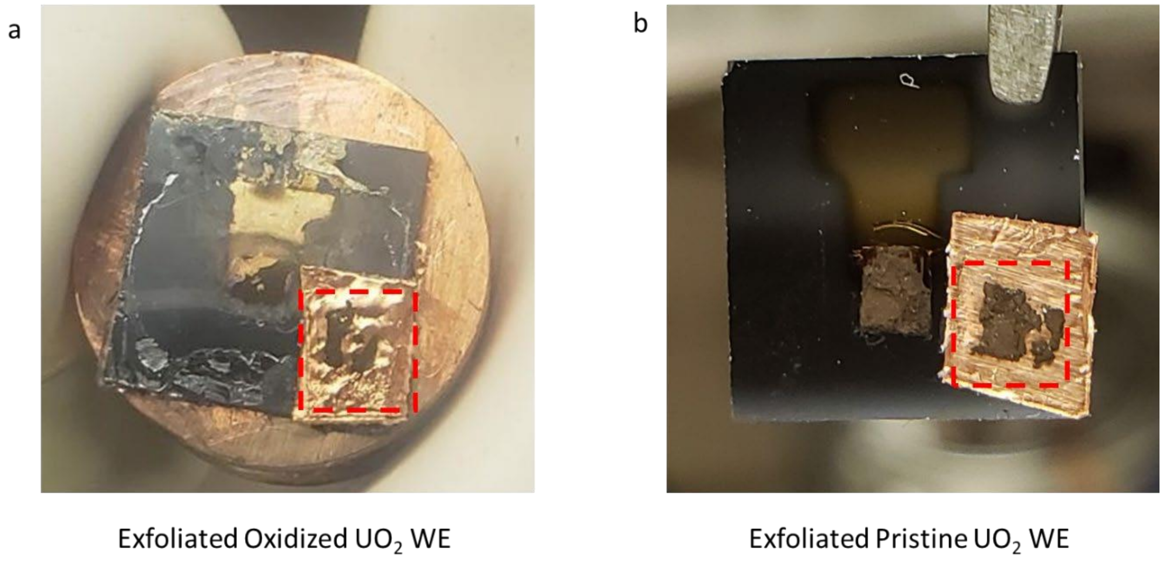

Figure S7. Photos of the exfoliated (a) oxidized and (b) pristine UO_2 WE surface. The red dashed squares indicate the XPS analysis areas.

Figure S7 shows the exfoliated surfaces of the oxidized and pristine UO_2 WE surface corresponding to XPS results shown in **Fig. 4** in the main text.

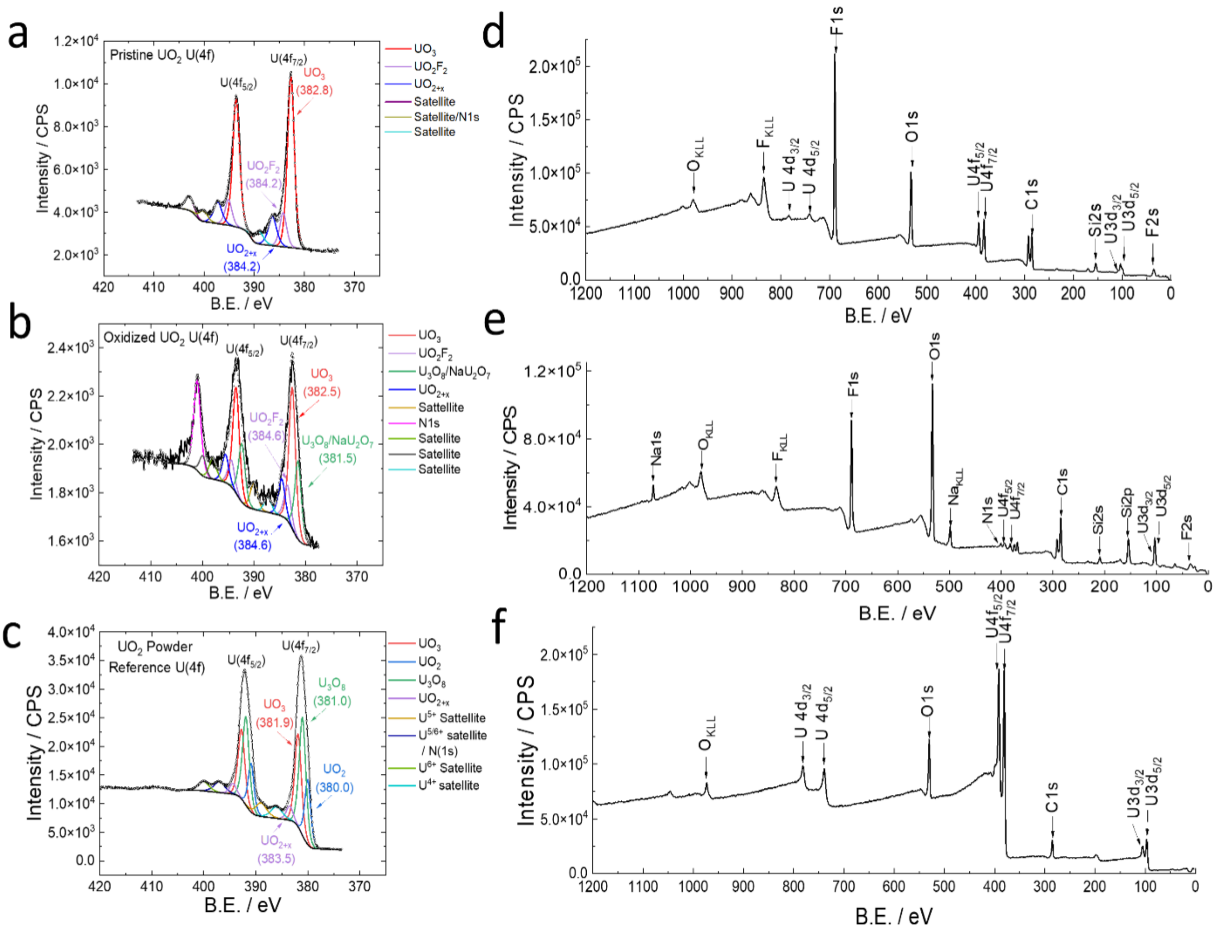

Figure S8. XPS spectral results for the narrow scan U(4f) region of (a) pristine UO_2 WE, (b) oxidized UO_2 WE, (c) and pristine UO_2 powder surfaces along with the corresponding wide scan survey plots. The U($4f_{7/2}$) peaks are identified with their respective binding energies with their doublet U($4f_{5/2}$) fixed at a difference of 10.9 eV due to spin-orbit coupling and a peak area ratio of 3:4 with the corresponding U($4f_{7/2}$).

The UO_2 powder narrow scan region (c) shows significant signals correspond to the oxidation of U (IV) to U(V) and U(VI), which is likely from air exposure over time. The pristine Nafion coated electrode shows the major species as UO_3 (69.7 at%), which is likely from further oxidation when exposed to the Nafion solution with non-stoichiometric UO_2 (14.1%) and UO_2F_2 (15.0%) as minor species present. After electrochemical processing, UO_3 is still present (71.1%) as well as minor contribution from U_3O_8/NaU_2O_7 (26.5%) and UO_2F_2 (12.1%).

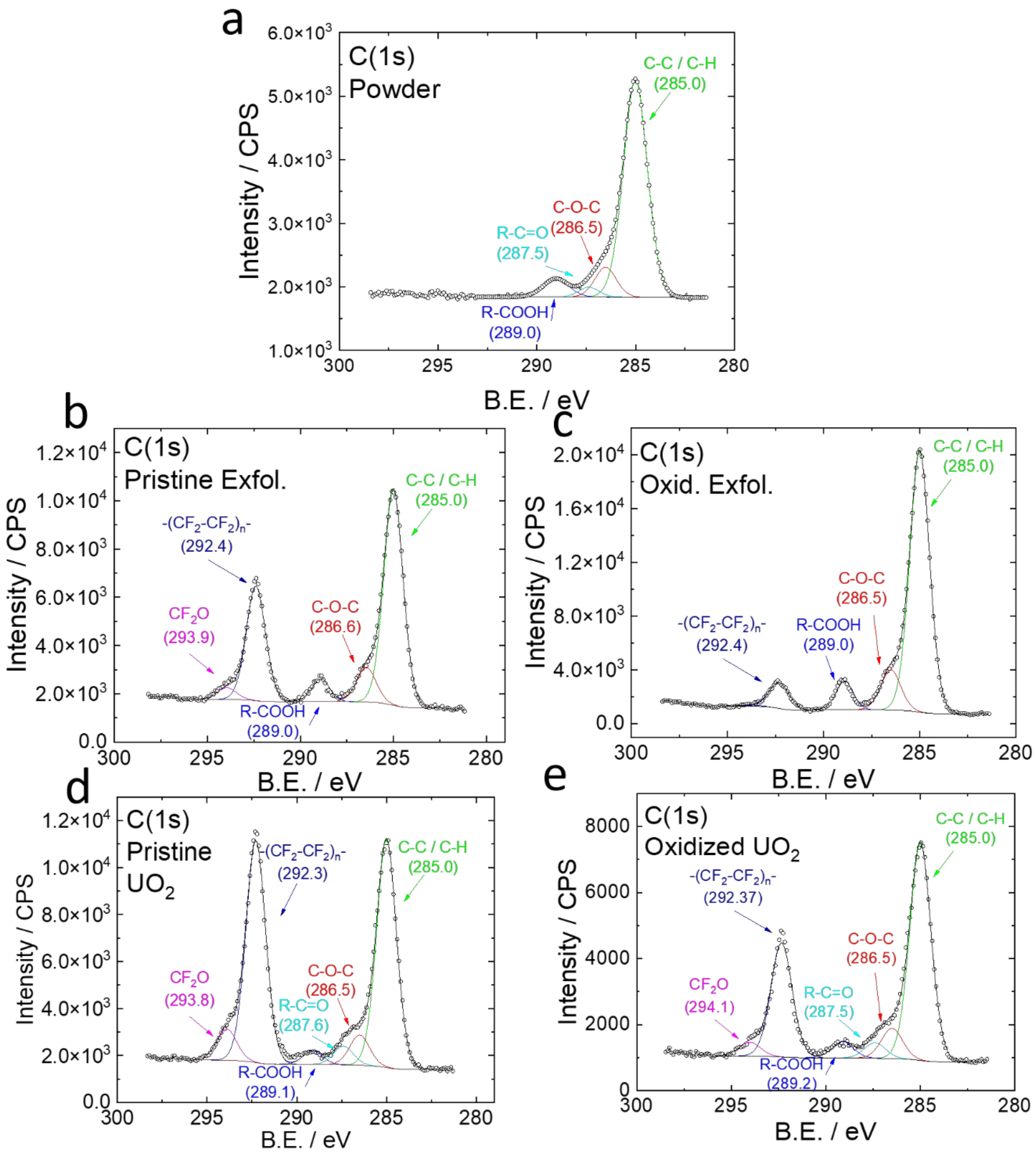

Figure S9. XPS narrow scan results for the C(1s) region for (a) powder UO_2 reference, (b) pristine and (c) oxidized exfoliated along with (d) pristine and (e) oxidized UO_2.

The XPS narrow scans for the C(1s) region are shown in **Fig. S6**. Significant Nafion presence is observed at 292.37 eV due to the $–(CF_2\text{-}CF_2)_n$ structure of Nafion. The Nafion peak decreases in intensity after electrochemical processing, which suggests that the membrane may be redistributed during potential sweeping [31].

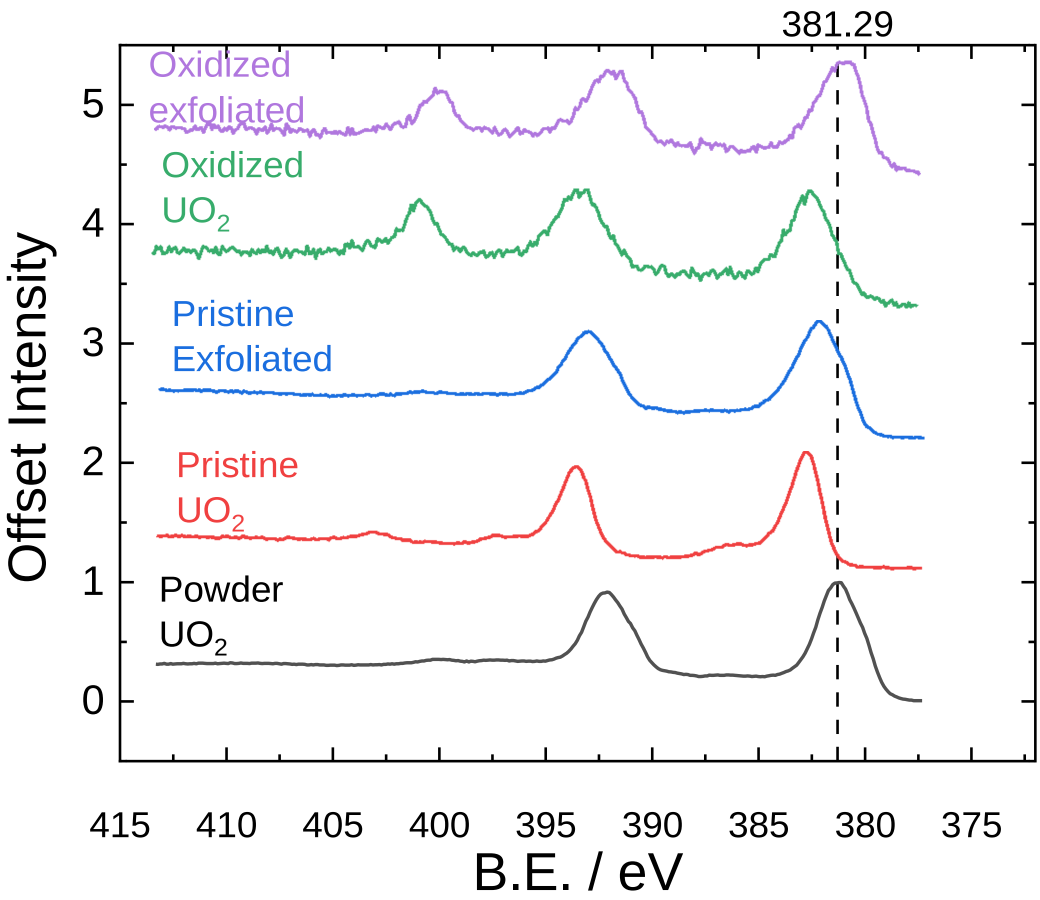

Figure S10. XPS spectral comparison of the U(4f) region for the electrodes with Powder UO_2 reference.

Powder UO_2 (which was also used to make the electrodes) was measured as a reference for the other UO_2 containing electrodes. When comparing the powder UO_2 to pristine UO_2 (contains a Nafion membrane), a peak shift to higher binding energies is observed [75]. This is likely due to the presence of Nafion, which may cause preferential charging (especially when present as an overlayer). Furthermore, after oxidation (both pristine and pristine exfoliated) shifts to a lower binding energy, which is consistent with some oxidation of the original surface and redistribution of the Nafion layer (thinner region for analysis, which results in less charging). Exact electrode preparation details are listed in Table S1 with atomic percent quantifications listed in Table S2, which were calculated using the U(4f) core level.

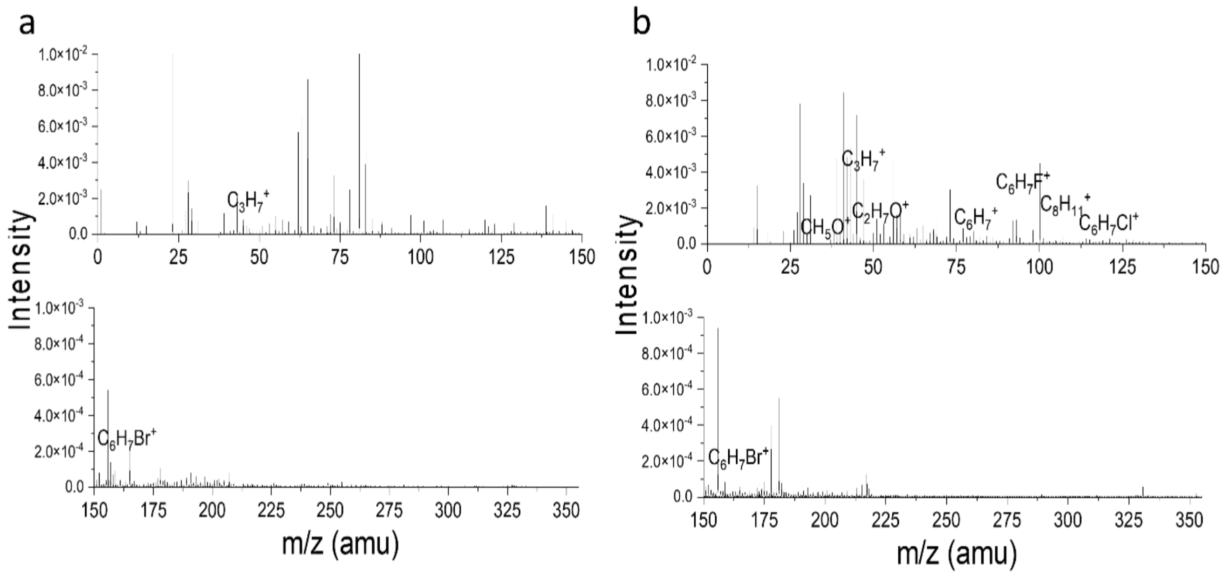

Figure S11. Normalized ToF-SIMS spectral comparison of the oxidized (a) and pristine (b) electrode surfaces.

Figure S11 depicts the ToF-SIMS spectral comparison of the pristine and electrochemically corroded electrode surfaces. Due to the instrument constraints to analyze uranium-containing samples, cerium oxide samples were used as surrogates. Because ToF-SIMS measurements are semiquantitative, the spectral data are normalized to total ion counts for ease of comparison. The relative lower counts of peaks related to Nafion (e.g., $C_3H_7^+$ m/z^+ 43.05 $C_8H_{11}^+$ m/z^+ 107.08, and $C_6H_7Br^+$ m/z^+ 157.97)[76] indicate that the surface counts of Nafion signals are less in the oxidized electrode than the pristine one. Also, the bumpier surface due to the protruding particles makes it difficult to get higher counts of ions in the extraction cone during measurements. The electrode morphological and topographic change can impact the spectral collection in surface measurements. The SIMS measurements were verified by the AFM topographic and amplitude measurements as shown in **Figs. 3b** & **3d** in the main text.

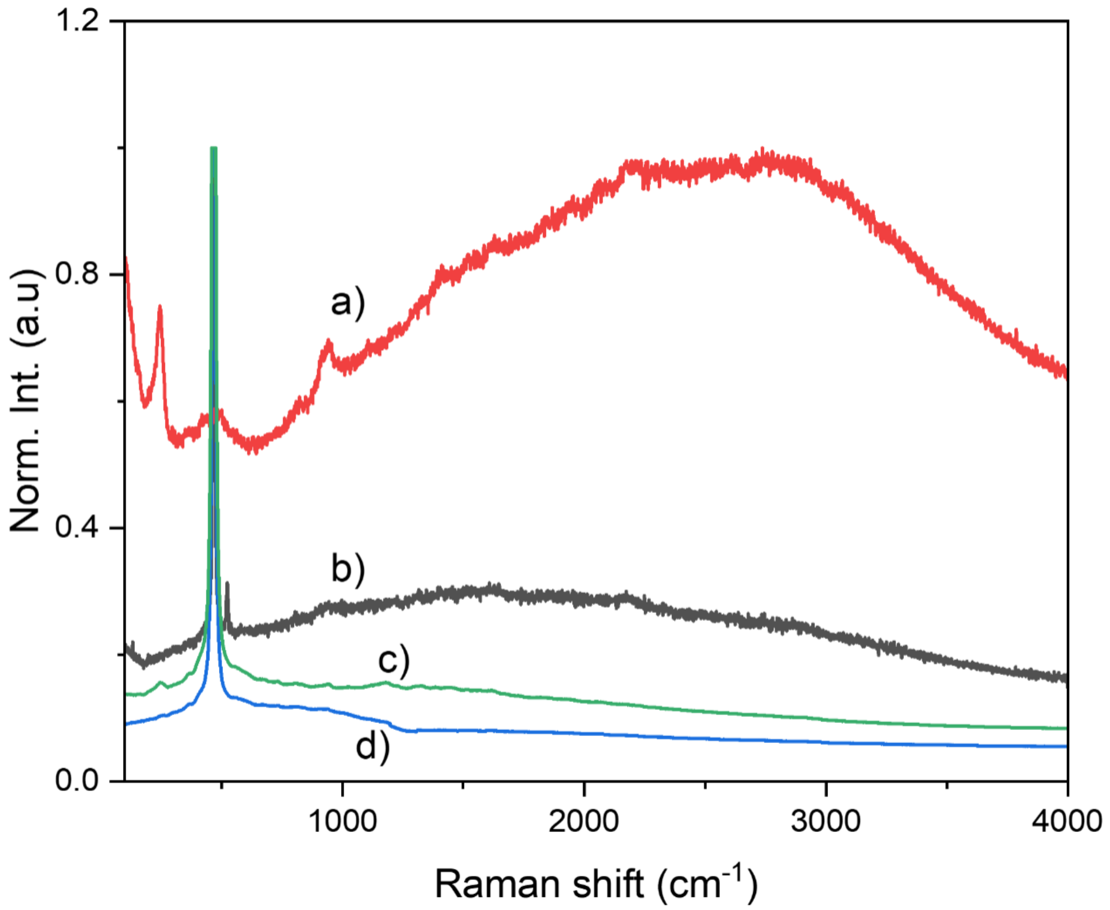

Figure S12. Raman spectral comparison of (a) freshly harvested corroded electrode from a SALVI E-cell device, (b) pristine electrode, (c) corroded electrode with water treatment, and (d) pristine electrode with water treatment.

Similarly, due to the instrument constraints to analyze uranium-containing samples, cerium oxide samples were used as surrogates. **Figure S12** depicts the Raman spectral comparison of the **freshly harvested electrode** after electrochemical corrosion, pristine as prepared electrode, a corroded electrode after being soaked in water, and a pristine electrode after being soaked in water. The Raman spectrum plots (**Figs. S12 a-d**) show the characteristic peak of CeO_2 at 462 cm^{-1}, verifying the existence of target particles. **Figure S12a** also shows a broad peak ranging from approximately 200 to 3500 cm^{-1}, which is different from the other electrode surfaces (**Figs. S12b, c, d**). This observation provides good evidence of the presence of interfacial water within the Nafion layer of the fabricated electrode. It indicates that the Nafion membrane could have absorbed some water from the aqueous electrolyte during the electrically driven corrosion process. **Figures S12c-d** show no sign of the water peak after the electrodes being soaked in water. It is worth noting that the peak shift in **Fig. S12a** is not as sharp as what would typically be observed of water (3000-~3600cm^{-1}) in Raman.

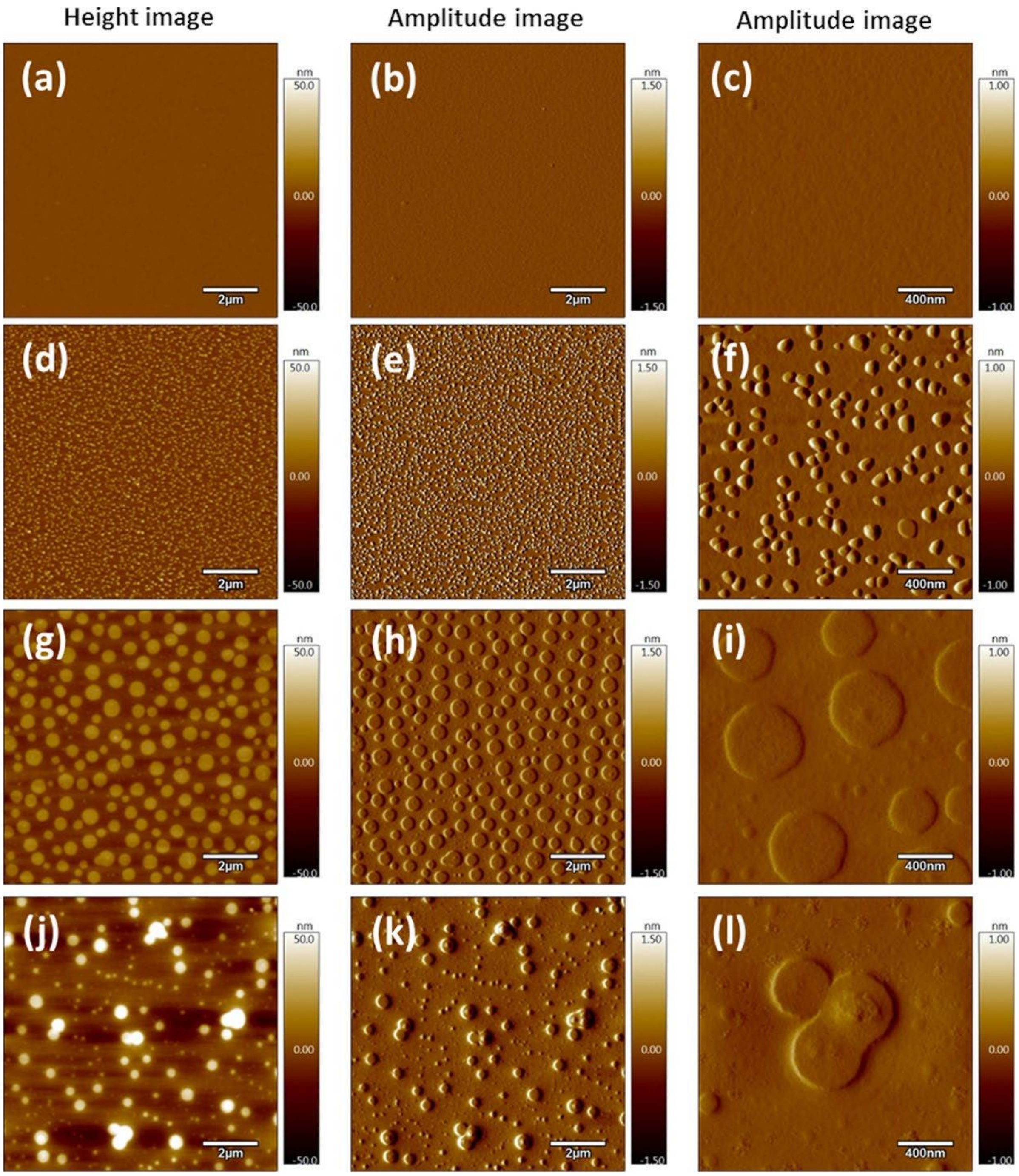

Figure S13. AFM height and amplitude images of ~35nm thick films of Nafion. (a-c) various conditions of Nafion and calix-2 with IECs 2.8 (d–f); 3.9 (g–i); and 5.8 (j–l). The scale bars are shown within the images.[77] by Chatterjee el. Al. JACS Au 2022, licensed under CC BY-NC-ND 4.0 ([https://pubs.acs.org/doi/10.1021/jacsau.2c00143?fig=fig4&ref=pdf)](https://pubs.acs.org/doi/10.1021/jacsau.2c00143?fig=fig4&ref=pdf)

Figure S13 (a, b, c) represent smooth surface of Nafion membrane by AFM imagining. This show default level smoothness of the Nafion membrane without any additive.

References

31. Son, J., E.C. Buck, S.L. Riechers, S. Tripathi, L.E. Strange, M.H. Engelhard, and X.Y. Yu, *Studying Corrosion Using Miniaturized Particle Attached Working Electrodes and the Nafion Membrane.* Micromachines (Basel), 2021. **12(11)**(11).
33. Liu, B., X.Y. Yu, Z. Zhu, X. Hua, L. Yang, and Z. Wang, *In situ chemical probing of the electrode-electrolyte interface by ToF-SIMS.* Lab Chip, 2014. **14**(5): p. 855-9.
35. Yu, J., Y. Zhou, X. Hua, S. Liu, Z. Zhu, and X.Y. Yu, *Capturing the transient species at the electrode-electrolyte interface by in situ dynamic molecular imaging.* Chem Commun (Camb), 2016. **52**(73): p. 10952-5.
55. Ilton, E.S. and P.S. Bagus, *XPS determination of uranium oxidation states.* Surf. Interface Anal., 2011. **43**(13): p. 1549-1560.
74. Son, J., E.C. Buck, S.L. Riechers, and X.Y. Yu, *Stamping Nanoparticles onto the Electrode for Rapid Electrochemical Analysis in Microfluidics.* Micromachines (Basel), 2021. **12(1)**(1).
75. Shutthanandan, V., M. Nandasiri, J. Zheng, M.H. Engelhard, W. Xu, S. Thevuthasan, and V. Murugesan, *Applications of XPS in the characterization of Battery materials.* J. Electron Spectrosc. Relat. Phenom., 2019. **231**: p. 2-10.
76. Gernatova, M., P. Janderka, A. Marcinkova, and P. Ostriz, *Use of Nafion as a membrane separator in membrane introduction of mass spectrometry.* Eur J Mass Spectrom (Chichester), 2009. **15**(5): p. 571-7.
77. Chatterjee, S., E. Zamani, S. Farzin, I. Evazzade, O.A. Obewhere, T.J. Johnson, V. Alexandrov, and S.K. Dishari, *Molecular-Level Control over Ionic Conduction and Ionic Current Direction by Designing Macrocycle-Based Ionomers.* JACS Au, 2022. **2**(5): p. 1144-1159.
